# Probing the Microheterogeneous Distribution of Photochemically Produced Hydroxyl Radicals in Dissolved Organic Matter

**DOI:** 10.1021/acs.est.5c13430

**Published:** 2025-12-17

**Authors:** Kai Cheng, Garrett McKay

**Affiliations:** Zachry Department of Civil & Environmental Engineering, 14736 Texas A&M University, College Station, Texas 77843, United States

**Keywords:** dissolved organic matter, hydroxyl radical, testosterone, microheterogeneous distribution, aliphaticity and aromaticity

## Abstract

Dissolved organic matter (DOM) is a major photosensitizer in sunlit surface waters that generates hydroxyl radicals (^•^OH). While ^•^OH is believed to form within hydrophobic DOM microdomains, its spatial distribution and phase-specific reactivity remain poorly characterized among DOM from diverse environments. In this study, we employed testosterone as a probe to quantify DOM-phase ^•^OH concentration ([^•^OH]_DOM_) via hydrophobic partitioning. Across eight DOM isolates, [^•^OH]_DOM_ was found to be 20–150 times higher than aqueous phase concentration ([^•^OH]_aq_). To explore the underlying drivers of this microheterogeneity, we evaluated [^•^OH]_DOM_/[^•^OH]_aq_ in relation to DOM composition. We report, for the first time, a negative correlation between [^•^OH]_DOM_/[^•^OH]_aq_ and aromaticity and a positive correlation with aliphaticity. Testosterone was further employed to quantify [^•^OH]_DOM_/[^•^OH]_aq_ for Suwannee River natural organic matter and humic acid isolates size fractionated with a 3 kDa ultrafiltration membrane. As expected, the <3 kDa showed little evidence of ^•^OH microheterogeneity. In contrast, the >3 kDa fraction showed lower [^•^OH]_DOM_/[^•^OH]_aq_ than the bulk fraction, suggesting that both high- and low-molecular weight components are important for the formation of microheterogeneous ^•^OH. Overall, these results establish the ubiquity of microheterogeneous ^•^OH formed during DOM photolysis and suggest that the abundance of aliphatic and aromatic carbon are important structural features governing this microheterogeneity.

## Graphical abstract

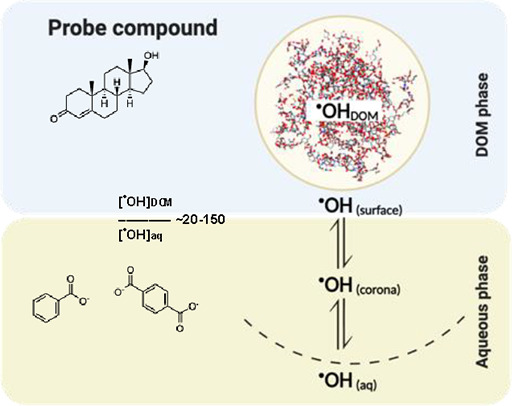

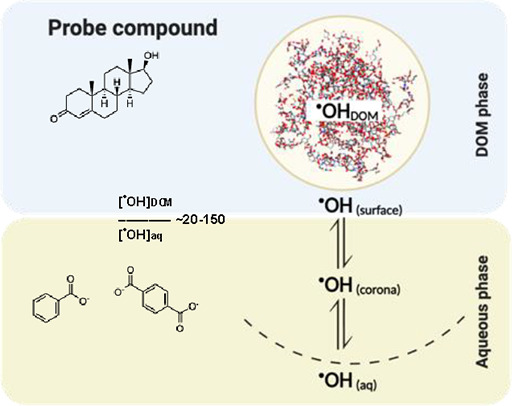

## 1 Introduction

Photochemically produced reactive intermediates (RIs) play a key role in environmental chemistry. In aquatic environments, the main source of RIs is typically the photolysis of dissolved organic matter (DOM), a complex and heterogeneous mixture of molecules.^1–3^ Although the exact structural configuration is not fully resolved, current models conceptualize DOM as a supramolecular assembly consisting of hydrophobic microenvironments within the interior of DOM aggregates and hydrophilic functional groups on the exterior.^4–7^ As a result, DOM displays microscopic heterogeneity, where hydrophobic regions may exclude solvents and act as microreactors.^8–10^ This microheterogeneity has been demonstrated for photochemical reactions of DOM, whereby concentrations of RIs including hydrated electron (e_aq_^–^),^11,12^ singlet oxygen (^1^O_2_),^13–18^ triplet excited state DOM (^3^DOM*),^19,20^ and hydroxyl radical (^•^OH)^21,22^ have been observed to be, in some cases, orders of magnitude higher in DOM microregions relative to the bulk, aqueous phase. Because most aquatic photochemistry studies employ probe compounds that measure only the aqueous phase concentration of these species,^23^ there exists the possibility that the importance of such species to environmental processes (e.g., DOM photooxidation and contaminant degradation) are underestimated.^10,16^

The microheterogeneous distribution of ^•^OH is of particular importance given its high reactivity with organic compounds. Recent studies have characterized the microheterogeneous distribution of ^•^OH using aminoglycoside antibiotics,^24^ cationic molecules that probe the surface region (i.e., corona), and chlorinated paraffins,^21^ hydrophobic molecules that probe DOM-phase ^•^OH. Results from these studies suggest that DOM-phase and surface ^•^OH concentrations are ∼200- and ∼15-fold higher than in the bulk, aqueous phase, respectively.^21,24^ However, several knowledge gaps still exist. First, the above-mentioned studies were performed exclusively with Suwannee River natural organic matter (SRNOM).^21,24^ Although SRNOM is a reliable surrogate for aquatic DOM, the extent of microheterogeneous ^•^OH production across different DOM sources is currently not known. Second, a limitation of employing chlorinated paraffins is that DOM-phase [^•^OH] measured were based on measurement of congeners, not individual isomers, which could impact the fundamental constants measured.^21^ Third, the transport of ^•^OH from the DOM-to aqueous phase is heavily influenced by quenching. As a result, DOM molecular size and composition will be important factors influencing the extent of ^•^OH scavenging in the DOM phase and thereby the amount of ^•^OH that can escape into the aqueous phase.^3,25–27^ A simple experiment to test this hypothesis would be to measure the microheterogeneous distribution of ^•^OH on size-fractionated DOM samples yet, to our knowledge, no such data exists in the literature for ^•^OH or other RI.

This study addresses the above-mentioned knowledge gaps by investigating the microheterogeneous distribution of ^•^OH for both a diverse collection of DOM isolates and selected isolates undergoing size fractionation. We first assessed the suitability of testosterone as a selective ^•^OH probe, followed by measuring apparent concentration of ^•^OH ([^•^OH]_app_) during photolysis of DOM solutions equilibrated with 10 μM testosterone. To ensure the generalizability of results, eight reference DOM isolates sourced from the International Humic Substances Society (IHSS)—spanning humic acid, fulvic acid, and reverse osmosis isolates from diverse environments—were employed. To quantify ^•^OH concentration in the DOM phase ([^•^OH]_DOM_), dialysis experiments were conducted to evaluate testosterone organic carbon–water partition coefficients (*K*_OC_). Two aqueous-phase probes, benzoate and terephthalate, were employed to quantify ^•^OH concentration in aqueous bulk phase ([^•^OH]_aq_). SRHA and SRNOM were fractionated using a 3 kDa ultrafiltration membrane to examine how molecular weight influences [^•^OH]_app_ measured by testosterone relative to aqueous phase probes.

## 2 Methods and Materials

### 2.1 Chemicals and Solution Preparation

DOM isolates were obtained from the IHSS, including Suwannee River humic acid (SRHA, 3S101H), Suwannee River fulvic acid (SRFA, 3S101F), SRNOM (2R101N), Upper Mississippi River natural organic matter (MRNOM, 1R110N), Pahokee Peat humic acid (PPHA, 1S103H), Pahokee Peat fulvic acid (PPFA, 2S103F), Elliott Soil humic acid (ESHA, 5S102H), and Elliott Soil fulvic acid (ESFA, 5S102F). DOM stock solutions (∼200 mg/L) were prepared by dissolving the solid isolate in Type I water produced from a Barnstead Nanopure purification system (Thermo Scientific, 18.20 MΩ cm resistivity). Details of solution preparation are included in the Supporting Information (Text S1).

Due to testosterone’s low aqueous solubility, cosolvents such as methanol or acetonitrile are often used to assist with dissolution. To eliminate potential ^•^OH quenching by cosolvents, testosterone stock solutions were prepared by stirring testosterone in water overnight. Undissolved solids were removed by filtration (0.45 μm poly­(ether sulfone), VWR). The stock solution concentration was determined by measuring the peak area with High Performance Liquid Chromatography (HPLC) and referencing an external calibration curve prepared using testosterone dissolved in methanol.^28^ Reported solubility limits for testosterone in water vary between 18 and 25 mg/L.^29,30^ In our study, the prepared stock solution had a measured aqueous concentration of 26.0 ± 0.5 mg/L. These discrepancies likely stem from differences in temperature and solution composition.

### 2.2 DOM Photolysis and Liquid Phase Extraction of Testosterone

Photolysis was conducted in uncapped borosilicate tubes placed in a Rayonet merry-go-round photoreactor fitted with mercury vapor lamps. The lamps emitted light with a peak (λ_max_) at 365 nm and a narrow bandwidth characterized by a full width at half-maximum (fwhm) of 18 nm (Figure S1). ^•^OH quantification was performed by irradiating solutions containing DOM (20 mg/L) and either testosterone (10 μM), benzoate (6.5 μM), or terephthalate (8.6 μM). Concentrations of the two aqueous phase probes (benzoate and terephthalate) were chosen to match the scavenging capacity of testosterone (*k*_2_^probe^[probe] = 3.8 × 10^4^ s^–1^), where *k*_2_^probe^ is the second order rate constant between the probe and ^•^OH. Matching *k*_2_^probe^[probe] ensured that bulk solution scavenging capacity was consistent across experiments with different probes, allowing differences in measured reactivity to be attributed to probe phase distribution rather than variations in ^•^OH concentration. Solutions were buffered with 10 mM phosphate, and pH was stable (7.0 ± 0.2) over the photolysis time scales employed. Prior to irradiation, solutions were stirred gently in the dark for 72 h to allow testosterone-DOM partitioning to reach equilibrium.

Photolysis experiments were conducted for 6 h in air-saturated solutions using uncapped tubes to allow air exchange. Evaporation-related mass loss was less than 3.5% by the end of the experiment (Figure S2). The reactor was equipped with cooling fans, which maintained the solution temperature at ∼33 °C (Figure S2). Testosterone concentration was measured using HPLC equipped with a UV and a fluorescence detector. Because HPLC analysis of aqueous solutions may fail to detect testosterone associated with the DOM phase (see Materials and Methods),^9^ liquid–liquid extractions were performed using diethyl ether.^30–32^ Preliminary experiments showed >95% recovery of testosterone from nonirradiated SRHA solution. Details of liquid–liquid extraction and HPLC analysis can be found in Texts S2 and S3, respectively.^9^ To ensure quantitation of DOM-bound testosterone, liquid–liquid extractions were performed using diethyl ether. To capture potential changes in recovery during irradiation, 17β-estradiol (octanol–water partitioning coefficient log *K*_ow_ = 3.10–4.01) was, spiked into solutions after irradiation as an extraction recovery surrogate, served as a surrogate to assess extraction recovery (Figure S3).^30^ Details of the liquid–liquid extraction and HPLC analysis can be found in Texts S2 and S3, respectively.

### 2.3 Dialysis Experiment and DOM Ultrafiltration

Equilibrium dialysis was used to quantify testosterone organic carbon–water partition coefficients (*K*_OC_, L/kg_C_). The experimental design was adapted from Christl et al.^33^ and Sibley and Pedersen.^34^ The Spectra/Por 7 tubing (1 kDa MWCO) was loaded with DOM solution and predialyzed for 24 h against 10 mM phosphate buffer; afterward, the buffer was replaced with testosterone solutions (1–50 μM) prepared in the same medium. Samples were shaken gently for 72 h to ensure equilibrium. *K*_OC_ were calculated for each DOM isolate according to a linear sorption model. Additional experimental steps and partitioning modeling details are provided in Texts S4 and S5, respectively.

Ultrafiltration was performed using a 200 mL UFSC20001 stirred cell (Amicon) and 3 kDa membrane, pressurizing the system with nitrogen gas (<75 psi). SRHA and SRNOM solutions with a volume of 200 mL (∼150 mg/L, pH 7) were filtered until 100 mL of permeate (batch one) was collected. To ensure more complete separation, the remaining retentate was diluted with 100 mL of Type I water and filtered again (batch two). Both retentate and permeate fractions (from two batches) were collected for analysis. Total organic carbon (TOC) was quantified using a TOC analyzer (TOC-L series, Shimadzu), and UV–visible spectra were recorded. The retentate and first permeate batch were subjected to photolysis experiments, while retentate fractions were also evaluated for testosterone interaction.

## 3 Results and Discussion

### 3.1 Characterization of Testosterone as an ^•^OH Probe Compound

To validate testosterone as an ^•^OH probe, it must be hydrophobic enough to partition into the DOM phase and should react selectively with ^•^OH. Testosterone’s hydrophobicity is well established, with a log *K*_ow_ = 3.22.^30^ Regarding selectivity, testosterone did not undergo direct photolysis under the employed irradiation conditions (Figure 1A), consistent with the slow phototransformation under natural sunlight (*t*_1/2_ = 6.4 d in summer at 40°N).^35,36^ As such, direct photolysis is not expected to be a major degradation pathway under the experimental conditions. In addition, testosterone was not degraded in the presence of 200 μM H_2_O_2_ in the dark. Testosterone’s stability under ^1^O_2_ exposure was verified using 10 μM perinaphthenone, with no observed degradation over 90 min of irradiation (Figure 1B). ^3^DOM* is also a potential oxidant that can react with testosterone and cause degradation. Given the heterogeneous composition of DOM, no single triplet energy value adequately describes it.^37^ To represent ^3^DOM* reactivity, we selected acetophenone (high triplet-state energy) and *p*-benzoquinone (high triplet-state one-electron reduction potential) as model ^3^DOM*. The degradation of 10 μM testosterone via these model triplets was minimal (2.6% loss by acetophenone and 1.7% loss by *p*-benzoquinone over 90 min). In contrast, testosterone degraded when irradiated in the presence of 200 μM H_2_O_2_ with a rate constant of 0.159 ± 0.008 h^–1^. These results show that testosterone reacts with ^•^OH but not ^1^O_2_ or model triplet sensitizers.

**Figure 1:**
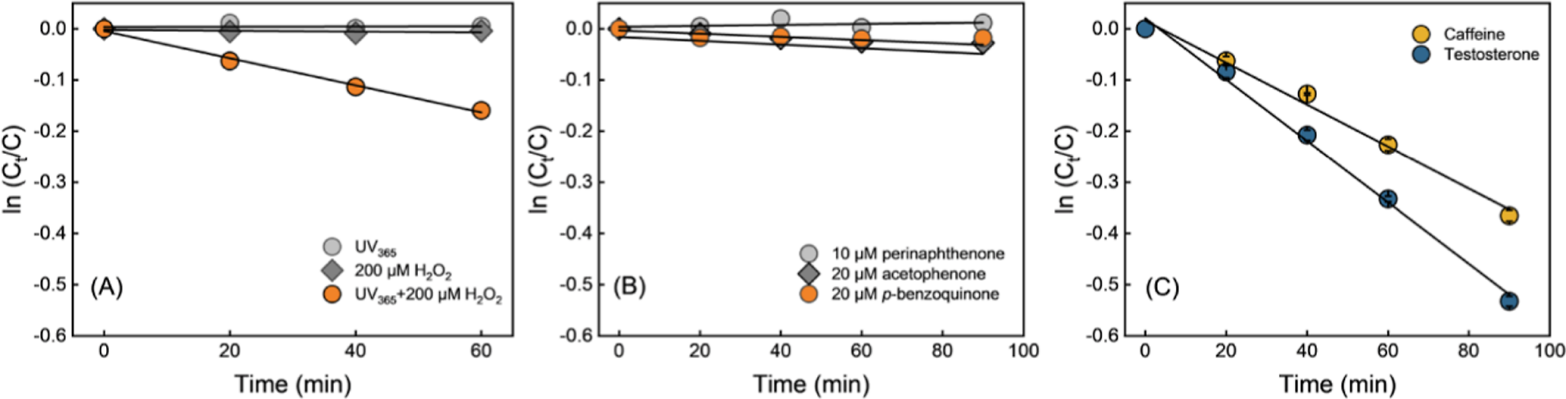
Assessment of testosterone as a probe for ^•^OH. (A) Photoreactivity under a narrow-band light source (λ_max_ = 365 nm, fwhm = 18 nm), including direct photolysis, dark control with 200 μM H_2_O_2_, and ^•^OH-mediated oxidation using 200 μM H_2_O_2_ as the ^•^OH source. (B) Reactivity with ^1^O_2_, sensitized by 10 μM perinaphthenone, and with triplet-state DOM model compounds of acetophenone and *p*-benzoquinone (each at 20 μM). (C) Determination of the second-order rate constant for testosterone with ^•^OH via competition kinetic against caffeine. H_2_O_2_ (1 mM) was employed as the ^•^OH source. All competition experiments were performed in triplicate.

We were unable to assess testosterone reactivity with hydrated electrons (e_aq_^–^), one-electron reductants (e.g., DOM^•–^), or singlet excited state DOM (^1^DOM*), all of which would be expected to be elevated in the DOM microenvironment. Another challenge is that we did not assess to what extent testosterone loss can distinguish between free ^•^OH and low-energy hydroxylating species. Thus, the role of such low-energy species in testosterone photodegradation is unclear.

Quantification of [^•^OH] using testosterone as a probe requires knowledge of its second-order rate constant (*k*_2_^TT^) with ^•^OH. *k*_2_^TT^ was measured via competition with caffeine, which has a well-characterized bimolecular rate constant of 2.6 × 10^9^ M^–1^ s^–1^.^38,39^ The reaction was performed using 1 mM H_2_O_2_ as the ^•^OH source under 365 nm photolysis. As shown in Figure 1C, under identical ^•^OH exposure conditions, testosterone degraded more rapidly than caffeine. The resulting *k*_2_^TT^ for testosterone was calculated as (3.81 ± 0.18) × 10^9^ M^–1^ s^–1^. This value falls within the range of reported steroid–^•^OH rate constants, such as progesterone (8.5 ± 0.9 × 10^8^ M^–1^ s^–1^) and estradiol (1.15 ± 0.28 × 10^10^ M^–1^ s^–1^).^40^ Similar to prior studies of ^•^OH microheterogeneity using aminoglycoside antibiotics and chlorinated paraffins,^41,42^ the [^•^OH]_app_ in this study was calculated using the ^•^OH bimolecular rate constant measured in the bulk phase. However, reactions between ^•^OH and DOM-bound testosterone may be better represented by a surface reaction instead of the bulk, aqueous-phase reaction rate constant. Future research is needed to evaluate whether probe compound adsorption to DOM surfaces impacts its bimolecular reaction rate constant with ^•^OH.

**Figure 2:**
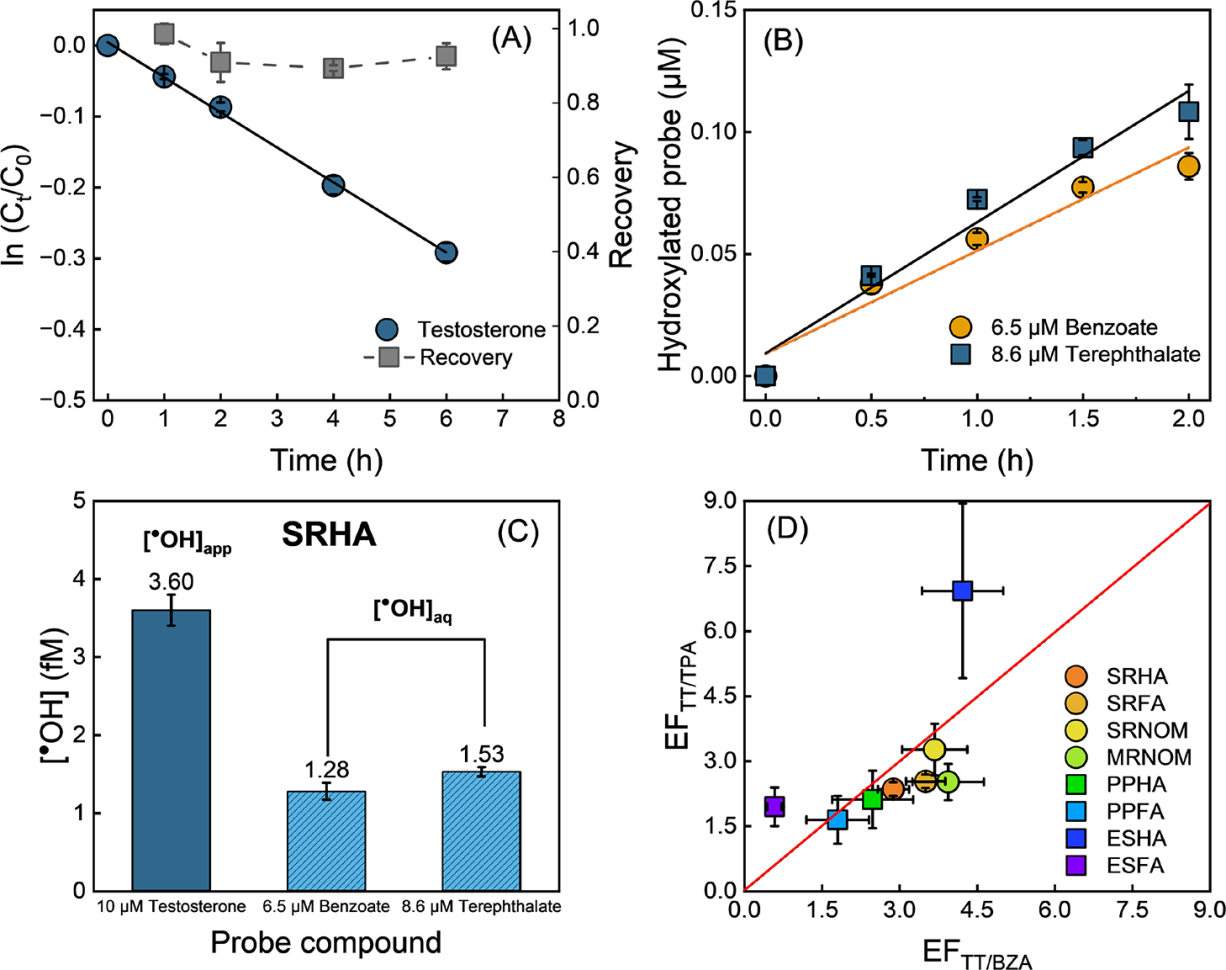
(A) Phototransformation of testosterone (10 μM) mediated by ^•^OH generated from SRHA photolysis. SRHA was used at 20 mg/L and buffered with 10 mM phosphate at pH 7. 17β-estradiol was applied as a surrogate to monitor liquid–liquid extraction recovery, shown as gray squares. (B) Formation of hydroxylated product of aqueous phase probe compound in reaction with ^•^OH. Salicylate and hydroxyterephthalate were monitored to quantify [^•^OH]_aq_. (C) Comparison of concentrations of ^•^OH measured by 10 μM testosterone, 6.5 μM benzoate, and 8.6 μM terephthalate. (D). Enhancement factor (EF = [^•^OH]_app_/[^•^OH]_aq_) calculated across various DOM isolates, based on the ratio of the concentration of ^•^OH measured by testosterone and those by aqueous-phase probes.

To validate the measured *k*_2_^TT^, nitrite was employed as a secondary ^•^OH source. A 230 μM nitrite solution was irradiated with 10 μM testosterone or 10 μM caffeine added independently. In this system, nitrite acts as both a source and sink.^43^ The calculated [^•^OH]_aq_ was 5.42 × 10^–14^ M from testosterone and 5.38 × 10^–14^ M from caffeine (Figure S4). The close agreement between the two values corroborates the measured *k*_2_^TT^.

### 3.2 Apparent ^•^OH Concentrations in DOM-Sensitized System

Testosterone (10 μM) was added as a probe compound in 20 mg/L DOM solutions and allowed to reach partitioning equilibrium over 72 h in the dark before initiating photolysis. By monitoring testosterone degradation, [^•^OH]_app_ can be determined accordingly (Figure S5). As shown in Figure 2A, the first order degradation rate constant of testosterone in SRHA solution was measured to be 0.049 ± 0.003 h^–1^, corresponding to an [^•^OH]_app_ of 3.6 ± 0.2 fM. Recovery of the extraction surrogate, 17β-estradiol, exceeded an average of 93%, suggesting an efficient extraction of testosterone from SRHA solution (extraction recoveries for all samples studied are shown in Supporting Information Figure S5). Both benzoate (6.5 μM) and terephthalate (8.6 μM) were used to probe [^•^OH]_aq._ These concentrations were employed to match the scavenging capacity of 10 μM testosterone to ensure identical [^•^OH]_ss_. The measured [^•^OH]_aq_ values of 1.2 ± 0.1 fM (benzoate) and 1.5 ± 0.1 fM (terephthalate) were two- to three-fold lower than [^•^OH]_app_ measured by testosterone (Figure 2C).

The enhanced [^•^OH]_app_ measured by testosterone relative to benzoate and terephthalate was examined for all DOM samples in this study (Figure 2D). The measured enhancement factors (EF = [^•^OH]_app_/[^•^OH]_aq_), which were calculated using both the benzoate and terephthalate probes, shows that ^•^OH microheterogeneity is a consistent feature across DOM isolates of diverse origins and extraction procedures. SRNOM, which contains a higher proportion of fulvic acid than humic acid, exhibited similar photochemical characteristics to SRFA but with a slightly increased EF ranging from 3.3 to 3.7. ESHA displayed the highest EF among the isolates, having values 4.2 and 6.9 for benzoate and terephthalate, respectively. In contrast, PPFA and ESFA, two soil fulvic acid isolates, showed the lowest EF values (≈2) of all the isolates. Overall, EF values calculated from benzoate and terephthalate were in good agreement, with ESHA being an exception.

Taken together, these results demonstrate that testosterone is exposed to a greater [^•^OH] than aqueous-phase probes, likely due to its partitioning into hydrophobic DOM domains (vide infra). Benzoate and terephthalate, both hydrophilic and negatively charged, are electrostatically repelled from the negatively charged DOM interface under neutral pH conditions, which limits their presence to the bulk aqueous phase. These results also align with the recent theoretical work by Mondino et al.^44^ that applied a two-phase reactivity model to suggest that DOM-phase partitioning can significantly influence the photodegradation of compounds with log *K*_ow_ > 3.

### 3.3 Calculation of DOM-phase [^•^OH]

As a hydrophobic probe, testosterone partitions into the DOM phase, and its degradation during photolysis reflects ^•^OH exposure in both environments. The DOM-bound fraction of testosterone reacts with ^•^OH near its sites of generation, while the freely dissolved fraction in the aqueous phase reacts with ^•^OH that has diffused from the DOM phase. Therefore, the observed degradation of testosterone represents contributions from both phases. The [^•^OH]_app_ experienced by testosterone can be described using a two-phase model,^13,16^ where [^•^OH]_app_ is the weighted sum of [^•^OH] in the DOM and aqueous phase (eq 1)­

[OH•]app=fDOM[OH•]DOM+faq[OH•]aq 1

where *f*_DOM_ and *f*_aq_ represent the fraction of testosterone in the DOM and the aqueous phase, respectively. *f*_DOM_ and *f*_aq_ were calculated using *K*_OC_, measured using equilibrium dialysis for each isolate. Sorption isotherms were derived from applying a series of increasing testosterone concentrations (1–50 μM) to 100 mg/L DOM solution. The equilibrium concentrations in water (*C*_w_, mol/L) and DOM (*C*_DOM_, mol/kg_C_) were fit to a linear sorption model (Figure S6).

Among the four aquatic DOM solutions tested (Figure 3A), testosterone exhibited the highest sorption to SRNOM with *K*_OC_ (L/kg_C_) being (6624 ± 698) and the lowest to SRFA with (3923 ± 559). Terrestrially derived isolates exhibited lower *K*_OC_ (L/kg_C_), with PPHA (4646 ± 1184) being the highest and PPFA, ESHA, and ESFA being more similar (*K*_OC_ ≈ 3500 L/kg_C_). The *K*_oc_ values measured here (log *K*_oc_ average of 3.64) are slightly lower than that reported previously by Neale et al. for Aldrich Humic Acid (log *K*_oc_ = 4.0).^45^ No significant relationships were observed in correlation plots of *K*_oc_ versus DOM physicochemical properties including elemental ratios and the content of aromatic, aliphatic, and phenolic moieties (Figure S7). This result is consistent with the mixed results reported in prior work: *K*_oc_ for estradiol was found to be within a narrow range for diverse DOM samples,^46^ while another study showed significant linear relationships between *K*_oc_ and phenolic content of DOM samples for both 17α- and 17β-estradiol.^47^

**Figure 3:**
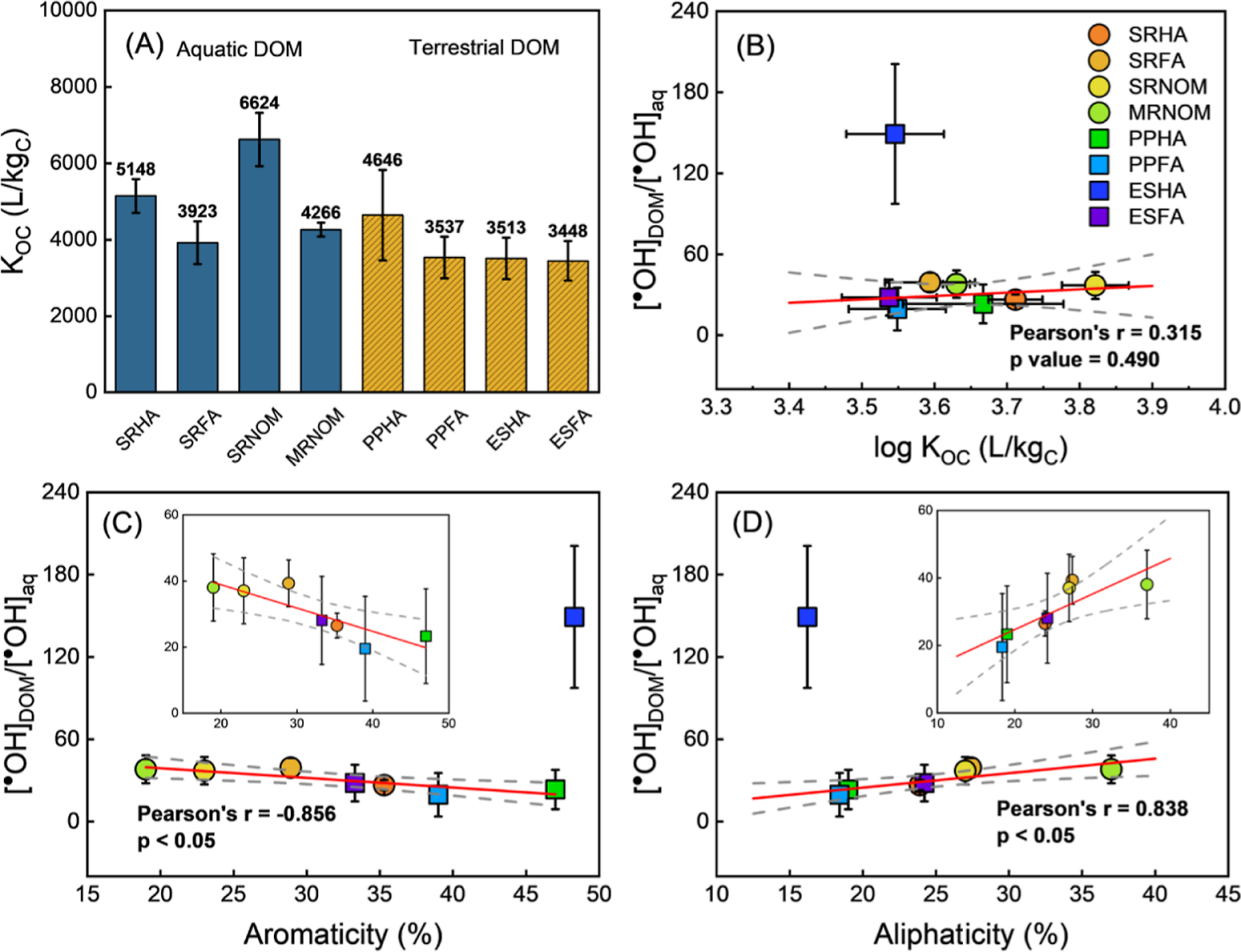
Testosterone partitioning behavior and its correlation with DOM composition and ^•^OH distribution. (A) Testosterone organic carbon-water partition coefficients (*K*_OC_, L/kg_C_) determined for eight DOM isolates using dialysis technique. (B) [^•^OH]_DOM_/[^•^OH]_aq_ values plotted against log*K*_OC_ (C). [^•^OH]_DOM_/[^•^OH]_aq_ values plotted against the aromaticity of DOM. (D) [^•^OH]_DOM_/[^•^OH]_aq_ values plotted against the aliphaticity of DOM. Compositional data (aromaticity and aliphaticity) for each DOM isolated were acquired from IHSS. [^•^OH]_DOM_/[^•^OH]_aq_ used here are the comparison of values derived from testosterone and terephthalate probes.

The experimentally determined *K*_OC_ values were used to calculate the fraction of testosterone partitioned into the DOM phase (Text S5). For SRNOM, the *f*_DOM_ of testosterone was calculated to be 0.063 ± 0.006 (*f*_DOM_ will be lower for other isolates due to lower *K*_oc_). If it is assumed that DOM-bound testosterone contributes less to ^•^OH quenching than DOM itself, then the [^•^OH]_aq_ in the aqueous phase can be approximated using values measured by benzoate and terephthalate (in the absence of testosterone). For SRNOM, applying the [^•^OH]_aq_ measured by benzoate to eq 1 yields a [^•^OH]_DOM_ of 41.8 ± 10.5 fM, and applying the [^•^OH]_aq_ measured by terephthalate resulted a similar [^•^OH]_DOM_ of 40.0 ± 10.5 fM. [^•^OH]_DOM_ represents the effective concentration of ^•^OH existing within DOM-associated microdomains prior to diffusion into bulk water, and reflects the average concentration across intra-DOM and interfacial regions determined from [^•^OH]_app_ measured by testosterone and the two-phase model.

The ratio of [^•^OH]_DOM_/[^•^OH]_aq_ serves as an intrinsic indicator of the importance of DOM-to aqueous-phase ^•^OH. For SRNOM, the measured [^•^OH]_DOM_/[^•^OH]_aq_ was 43.6 ± 11.0 (by benzoate) and 37.1 ± 10.0 (by terephthalate). Our value of [^•^OH]_DOM_/[^•^OH]_aq_ for SRNOM determined using testosterone is lower than that reported recently by Yan et al.^21^ ([^•^OH]_DOM_/[^•^OH]_aq_ of 210 ± 31), who used chlorinated paraffins and a solar simulator. Our [^•^OH]_DOM_/[^•^OH]_aq_ is more closely aligned to the value measured by Li et al.^41^ ([^•^OH]_DOM_/[^•^OH]_aq_ ≈ 38), where [^•^OH]_DOM_ represents the “surface” concentration measured using (cationic) aminoglycoside antibiotics. Our results of [^•^OH]_DOM_/[^•^OH]_aq_ in comparison to literature are reasonable given the multiple sources of uncertainty in prior studies (e.g., use of chlorinated paraffin congeners) and this work (potential for e_aq_^–^ and DOM^•–^ degradation of testosterone). Further improvement in accurate quantification of [^•^OH]_DOM_/[^•^OH]_aq_ could be made by employing probe compounds more hydrophobic than testosterone. For example, the fraction of bound testosterone is overall quite low (*f*_DOM_ ∼0.05), which limits its sensitivity for quantification of [^•^OH]_DOM_. Ideally, such a probe compound would also form ^•^OH-specific degradation products that could be tracked.

### 3.4 ^•^OH Microheterogeneity in Diverse DOM Isolates

Little information exists on the ubiquity of ^•^OH microheterogeneity across diverse DOM samples. Here, we show that the [^•^OH]_DOM_/[^•^OH]_aq_ measured using testosterone range from ∼20 to 150 for DOM across diverse origins (soil vs aquatic) and extraction procedures. Although benzoate- and terephthalate-derived [^•^OH]_aq_ values varied slightly, the calculated [^•^OH]_DOM_/[^•^OH]_aq_ were largely consistent across the isolates studied (Table 1). Excluding ESHA, the range of [^•^OH]_DOM_/[^•^OH]_aq_ was ∼20–70 across the two aqueous-phase probe measurements. SRFA, SRNOM, and SRHA showed comparable [^•^OH]_DOM_/[^•^OH]_aq_ values, with some variation observed based on the aqueous probe choice. Interestingly, SRHA’s [^•^OH]_DOM_/[^•^OH]_aq_ values are on the lower end of the range for the Suwannee River isolates. ESHA exhibited the highest [^•^OH]_DOM_/[^•^OH]_aq_ value (149.1 ± 51.7) among the studied samples, but the corresponding fulvic acid extract ESFA had one of the lowest (28.0 ± 13.0). In contrast, [^•^OH]_DOM_/[^•^OH]_aq_ for PPHA and PPFA were more similar. Aquatically derived isolates exhibited a similar range of [^•^OH]_DOM_/[^•^OH]_aq_ as soil-derived isolates (apart from ESHA). These comparisons illustrate that qualitative variables such as isolation procedure or DOM origin are not good predicators of [^•^OH]_DOM_/[^•^OH]_aq_.

**Table 1:** Summary of ^•^OH Concentrations, Testosterone Partitioning Across DOM Phases, and Calculated [^•^OH]_DOM_/[^•^OH]_aq_ Ratios

	photolysis kinetics			testosterone sorption		intra-DOM phase		microheterogeneity	
DOM	[^•^OH]_app_ fM	[^•^OH]_aq_ fM		*K*_OC_ (L/kg_C_)	TT partitioning^a^	[^•^OH]_DOM_ fM		[^•^OH]_DOM_/[^•^OH]_aq_	
	TT	BZA	TPA		*f*_DOM_	BZA	TPA	BZA	TPA
SRHA	3.60 ± 0.20	1.28 ± 0.11	1.53 ± 0.06	5148 ± 441	0.053 ± 0.005	45.6 ± 6.1	40.6 ± 5.5	36.5 ± 5.8	26.5 ± 3.7
SRFA	3.47 ± 0.21	0.99 ± 0.09	1.37 ± 0.02	3922 ± 559	0.040 ± 0.006	63.0 ± 11.0	53.9 ± 9.7	63.6 ± 12.5	39.3 ± 7.1
SRNOM	3.53 ± 0.60	0.96 ± 0.02	1.08 ± 0.07	6624 ± 698	0.063 ± 0.006	41.8 ± 10.5	40.0 ± 10.5	43.6 ± 11.0	37.1 ± 10.0
MRNOM	3.15 ± 0.50	0.80 ± 0.06	1.25 ± 0.06	4266 ± 182	0.041 ± 0.002	58.1 ± 12.6	47.6 ± 12.5	72.6 ± 16.7	38.1 ± 10.2
PPHA	3.64 ± 1.09	1.47 ± 0.15	1.72 ± 0.16	4646 ± 1184	0.050 ± 0.013	44.9 ± 24.9	40.1 ± 24.4	30.5 ± 17.2	23.3 ± 14.3
PPFA	3.66 ± 1.20	2.03 ± 0.15	2.22 ± 0.14	3536 ± 544	0.035 ± 0.005	48.6 ± 35.2	43.4 ± 35.1	23.9 ± 17.4	19.5 ± 15.8
ESHA	4.64 ± 0.86	1.10 ± 0.02	0.67 ± 0.15	3513 ± 543	0.040 ± 0.006	89.6 ± 25.4	99.9 ± 26.5	81.5 ± 23.1	149.1 ± 51.7
ESFA	2.59 ± 0.59	4.43 ± 0.20	1.33 ± 0.02	3448 ± 517	0.035 ± 0.005	NA	37.3 ± 17.7	NA	28.0 ± 13.0

a TT represents testosterone that was added to DOM solutions at a concentration of 10 μM. BZA and TPA represent benzoate (6.5 μM) and terephthalate (8.6 μM) selected to quantify the concentration of ^•^OH in aqueous phase. The concentrations were chosen to contribute the same scavenging capacity as 10 μM testosterone to ensure a similar [^•^OH]_ss_ level. *f*_DOM_ represents the fraction of testosterone partitioned into the DOM phase at added DOM concentration calculated from *K*_OC_. [^•^OH]_DOM_ was calculated using eq 1.

To further investigate ^•^OH microheterogeneity, correlation analysis was conducted between [^•^OH]_DOM_/[^•^OH]_aq_ values determined from testosterone and terephthalate probes with DOM compositional characteristics reported by the International Humic Substances Society (Figure 3C,D). ESHA was excluded from this analysis because its [^•^OH]_DOM_/[^•^OH]_aq_ was an outlier. Pearson correlation analysis was used because the relationships between variables appeared approximately linear, and inspection of residuals showed no systematic deviation from linearity or normality. The analysis revealed a significant negative correlation (|*r*| > 0.8, *p* < 0.05) with aromaticity and a positive correlation with aliphaticity, indicating that [^•^OH]_DOM_/[^•^OH]_aq_ increases with increasing aliphatic carbon content. Correlations using benzoate as an aqueous phase probe are qualitatively similar (Figure S8).

The observed relationships between [^•^OH]_DOM_/[^•^OH]_aq_ with aliphatic and aromatic content is, to our knowledge, the first reported correlations linking DOM composition to ^•^OH microheterogeneity. Because aliphatic moieties are not expected to generate ^•^OH under UV-A irradiation, the positive correlation with aliphatic content could potentially reflect structural effects. Inspired by Perminova et al.,^48^ we hypothesize that aliphatic groups act as spacers between chromophores within DOM assemblies, increasing the distance ^•^OH radicals need to diffuse (and thus the potential for quenching) before reaching the bulk aqueous phase. Such increased quenching in effect would lower [^•^OH]_aq_ in greater proportion relative to [^•^OH]_DOM_, resulting in an increased [^•^OH]_DOM_/[^•^OH]_aq_. Consistent with this hypothesis, Grandbois et al.^13^ reported a larger DOM radius for Pony Lake fulvic acid (1.7–5.8 nm) relative to SRHA (0.65–2.2 nm), with the former having more than 2-fold higher aliphatic carbon content as measured by ^13^C NMR. Taken together, these findings suggest that DOM aromaticity and aliphaticity may be playing a role in shaping the DOM microenvironments that govern the extent of ^•^OH microheterogeneity. Because this conclusion is based on correlation analysis, future studies should be conducted to provide more direct experimental evidence.

### 3.5 Molecular Weight-Dependent ^•^OH Microheterogeneity

DOM can be envisioned as a supramolecular assembly of molecules with varying sizes, stabilized by hydrophobic interactions and hydrogen bonding.^4–7,49–51^ Molecular weight is a key factor influencing DOM properties,^52–54^ but its impact on the microheterogeneous distribution of RI is unclear. Here, we separated SRHA and SRNOM into high and low molecular weight fractions using a 3 kDa ultrafiltration membrane. Testosterone was employed to measure [^•^OH]_app_ in both the low (<3 kDa) and high (>3 kDa) molecular weight fraction. *K*_OC_ values were also measured for the high molecular fraction to enable calculation of [^•^OH]_DOM_. We hypothesized that the >3 kDa fraction would exhibit higher [^•^OH]_DOM_/[^•^OH]_aq_ than the bulk sample and that the <3 kDa fraction would be lower than the bulk. *K*_OC_ for the >3 kDa SRHA fraction (Figure S9) was measured as 7132 ± 1047 L/kg_C_, which is 1.38 times greater than that of unfractionated SRHA (5148 ± 441 L/kg_C_). The first-order rate constant for testosterone photodegradation in the >3 kDa SRHA fraction (10 mg_C_/L) was measured to be 0.054 ± 0.006 h^–1^, corresponding to an [^•^OH]_app_ of 3.9 ± 0.4 fM. Although this value is statistically indistinguishable from the unfractionated SRHA ([^•^OH]_app_ of 3.6 ± 0.2 fM), the [^•^OH]_aq_ of bulk SRHA was lower than the >3 kDa fraction, resulting in [^•^OH]_DOM_/[^•^OH]_aq_ being ∼30% lower in the >3 kDa SRHA fraction (Figure 4B). Likewise, the [^•^OH]_DOM_/[^•^OH]_aq_ determined using terephthalate for the >3 kDa was indistinguishable from bulk SRHA. Considering the <3 kDa fraction (3.0 mg_C_/L), there was no apparent difference in [^•^OH ]_app_ measured by testosterone and either benzoate or terephthalate. This could be due to the low sensitivity of testosterone (low *f*_DOM_) or the lack of microheterogeneous ^•^OH distribution in the <3 kDa fraction. For SRNOM, *K*_OC_ for the >3 kDa fraction (9240 ± 506 L/kg_C_) was 1.40 times greater than for unfractionated SRNOM (6624 ± 698 L/kg_C_). The [^•^OH]_app_ measured by testosterone in the >3 kDa SRNOM fraction was 3.16 ± 0.09 fM, corresponding to a [^•^OH]_DOM_/[^•^OH]_aq_ of 22.7 ± 1.6 and 24.8 ± 1.7 for benzoate and terephthalate, respectively. This is about half the ratio observed in the unfractionated SRNOM solution (37.1 ± 10.0 to 43.6 ± 11.0). In the <3 kDa fraction of SRNOM (8.2 mg_C_/L), photolysis experiments showed comparable [^•^OH]_app_ measured by testosterone (0.48 ± 0.15 fM) and aqueous probes (0.48 and 0.60 fM), indicating no evidence of phase-specific enhancement.

**Figure 4:**
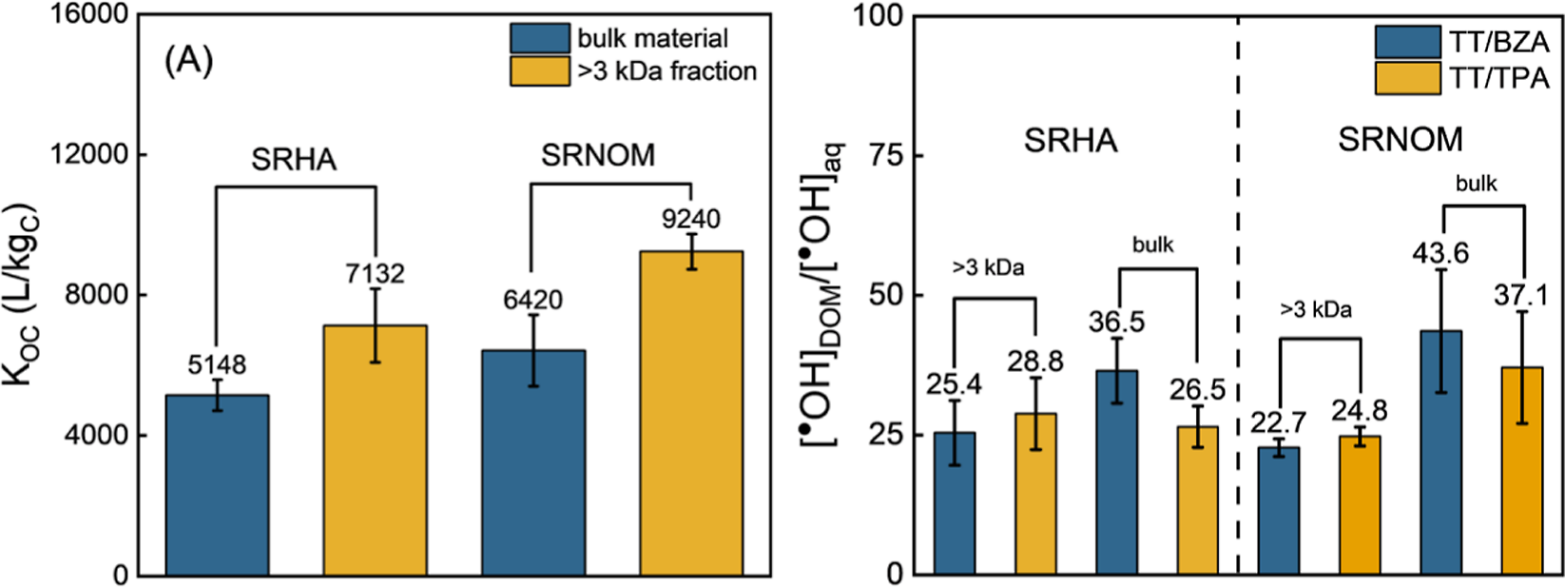
(A) Sorption coefficient *K*_OC_ (L/kg_C_) of testosterone to the high molecular weight (>3 kDa) DOM fractions of SRHA and SRNOM compared with those of the unfractionated bulk materials. (B) [^•^OH]_DOM_/[^•^OH]_aq_ ratios calculated for the bulk and >3 kDa fractions of SRHA and SRNOM. TT/BZA and TT/TPA refer to values derived using testosterone as the DOM-phase probe in combination with benzoate or terephthalate, respectively, as the aqueous-phase probe.

The results from molecular weight fractionation of SRHA and SRNOM are contrary to our initial hypothesis that the >3 kDa fraction would have enhanced [^•^OH]_DOM_/[^•^OH]_aq_ relative to the bulk sample. Instead, both fractions have lower [^•^OH]_DOM_/[^•^OH]_aq_ than the bulk sample, with the <3 kDa fraction showing no evidence of ^•^OH microheterogeneity ([^•^OH]_DOM_/[^•^OH]_aq_ ≈1). We do not have a simple explanation for this observation. One possibility is that both high- and low molecular weight components are involved in DOM supramolecular assemblies responsible for the microheterogeneous effect. Another could be that ultrafiltration not only separates DOM by size but also produces distinct chemical compositions in the resulting fractions. Although the lack of clarity regarding the mechanism is unsatisfying, we hope these results will inspire research into RI microheterogeneity in DOM molecular weight fractions.

## 4 Environmental Implication

Enhanced level of RIs in interfacial regions has been widely reported as a key feature of water chemistry. Such phenomena occur not only at the interface of DOM and water but also at the air–water,^55,56^ mineral–organic,^57^ sediment–water, and ice–water^58^ interfaces because of their unique thermodynamic and kinetic properties, including species enrichment and localized oxygen availability.^55,56^ Numerous prior studies have documented the microheterogeneous distribution of ^1^O_2_ in DOM-containing systems, yet investigations of ^•^OH have been limited to a few studies focused on SRNOM. Expanding on this knowledge base, our work employed testosterone as a hydrophobic probe to assess ^•^OH microheterogeneity across a broader suite of DOM isolates. DOM-phase ^•^OH concentrations were found to be 20–150 times higher than those in the aqueous phase, demonstrating that such microheterogeneity is a generalizable feature of diverse DOM types. For SRNOM, specifically, [^•^OH]_DOM_/[^•^OH]_aq_ values ranged from 37.1 to 43.6, which falls within the lower-middle portion of the previously reported range (16–210),^21^ and closely matches the value derived from cationic compound probed near-surface and aqueous-phase [^•^OH] ratio of 38.4.^24^ Our findings provide new opportunities for further investigation of how DOM composition shapes ^•^OH microheterogeneity.^21^ Specifically, we observed a positive correlation between [^•^OH]_DOM_/[^•^OH]_aq_ and DOM aliphaticity, and a negative correlation with aromaticity. This suggests that aliphatic-rich DOM matrices promote enhanced internal ^•^OH quenching, possibly due to steric effects between DOM chromophores and expansion of hydrophobic domains, lowering [^•^OH]_aq_. It is important to note that the suggested role of aliphatic domains in quenching ^•^OH is based on correlation (Figure 3) and inference from size-fractionated samples (Table 2). Future studies employing additional methods such as radical spin trapping could provide additional testing of this hypothesis.

**Table 2:** Organic Carbon, UV–Visible Spectroscopy, Molecular Weight Fractions, and Their Production of ^•^OH Radical

SRHA	mass balance (mg_C_)	E2/E3	SUVA_254_ (L/mg_C_·m)	*K*_OC_ (L/kg_C_)	[^•^OH]_DOM_/[^•^OH]_aq_ ^a^	
					BZA	TPA
bulk	15.90	3.42	4.96	5148 ± 441	36.5 ± 5.8	26.5 ± 3.7
>3 kDa	14.64	3.37	6.01	7132 ± 1047	25.4 ± 5.8	28.8 ± 6.4
<3 kDa	0.59	4.25	3.06	—	—	—

SRNOM	mass balance (mg_C_)	E2/E3	SUVA_254_ (L/mg_C_·m)	$K_{OC}\left(\frac{L}{kg\cdot_{C}}\right)$	[^•^OH]_DOM_/[^•^OH]_aq_ ^a^	
					BZA	TPA
bulk	17.50	4.66	3.25	6624 ± 698	43.6 ± 11.0	37.1 ± 10.0
>3 kDa	14.43	4.57	4.20	9240 ± 506	22.7 ± 1.6	24.8 ± 1.7
<3 kDa	1.83	5.97	2.30	—	—	—

a BZA and TPA represent benzoate (6.5 μM) and terephthalate (8.6 μM) selected to quantify the concentration of ^•^OH in aqueous phase.

The influence of DOM binding on photochemical pathways can differ substantially when compared to homogeneous solutions. Prior studies illustrate two extremes: in the case of mirex photoreduction,^11,12^ the transformation is largely confined to the DOM-bound phase, whereas compounds that remain predominantly in the aqueous phase show limited access to phase-specific reactivity occurring within DOM microdomains. These contrasting behaviors highlight that the fate of organic contaminants depends strongly on their phase distribution. Therefore, evaluating the extent of DOM microheterogeneity using the ratio of [RI]_DOM_ to [RI]_aq_ provides meaningful insight for compounds that associate with DOM in sunlit waters, where localized reactivity may control transformation. Understanding and quantifying these phase-dependent photoreactions is therefore critical for predicting contaminant persistence, reactivity hotspots, and the formation of byproducts under environmentally relevant conditions. Due to the focus on a large number of isolate materials, we did not explore testosterone sorption at higher DOM concentrations, which would have led to an increased fraction of testosterone in the DOM phase and consequently a greater sensitivity in our ability to quantify [^•^OH]_DOM_. This issue could be addressed in future studies by increasing DOM concentrations. Perhaps a more productive avenue for future research is identification of more hydrophobic probes that partition even more strongly into DOM domains without significantly changing DOM structure. Such probes would enable a more sensitive assessment of oxidative processes occurring within DOM microdomains.

Models for DOM photochemistry largely rely on bulk-phase concentrations of reactive intermediates, potentially underestimating degradation rates in natural systems by neglecting microheterogeneous reactive species. Due the difficulty in measuring these distributions in each unique situation, one path forward is to increase the number of laboratory determinations while also increasing our understanding of the DOM compositional factors that influence this microheterogeneity. The present work contributes toward this goal by providing an experimental data set that can be used to enhance the spatial resolution of photochemical models and demonstrating a dependence on DOM structure.

## Supplementary Material



## Notes

Supporting Information provides descriptions of analytical methods, kinetic and sorption modeling, dialysis and ultrafiltration experiments details, and a complete list of chemicals used. Additional data include the UV lamp spectrum, validation of testosterone reaction kinetics with hydroxyl radicals, and experiments on testosterone photodegradation and sorption in various DOM isolates (PDF)

## References

[1] Aiken G. R., Hsu-Kim H., Ryan J. N. (2011). Influence of Dissolved Organic Matter
on the Environmental Fate of Metals, Nanoparticles, and Colloids. Environ. Sci. Technol..

[2] Zark M., Dittmar T. (2018). Universal Molecular Structures in Natural Dissolved
Organic Matter. Nat. Commun..

[3] Vione D., Falletti G., Maurino V., Minero C., Pelizzetti E., Malandrino M., Ajassa R., Olariu R. I., Arsene C. (2006). Sources and
Sinks of Hydroxyl Radicals Upon Irradiation of Natural Water Samples. Environ. Sci. Technol..

[4] Piccolo A. (2001). The Supramolecular
Structure of Humic Substances. Soil Sci..

[5] Sutton R., Sposito G. (2005). Molecular Structure
in Soil Humic Substances: The New
View. Environ. Sci. Technol..

[6] Wells M. J., Stretz H. A. (2019). Supramolecular Architectures of Natural Organic Matter. Sci. Total Environ..

[7] Norris K. E., Pignatello J. J., Vialykh E. A., Sander M., McNeill K., Rosario-Ortiz F. L. (2025). Recent Developments on the Three-Dimensional Structure
of Dissolved Organic Matter: Toward a Unified Description. Environ. Sci. Technol..

[8] Vialykh E. A., McKay G., Rosario-Ortiz F. L. (2020). Computational Assessment of the Three-Dimensional
Configuration of Dissolved Organic Matter Chromophores and Influence
on Absorption Spectra. Environ. Sci. Technol..

[9] Hassett J. P., Anderson M. A. (1979). Association of Hydrophobic
Organic-Compounds with Dissolved
Organic-Matter in Aquatic Systems. Environ.
Sci. Technol..

[10] Hassett J. P. (2006). Chemistry
- Dissolved Natural Organic Matter as a Microreactor. Science.

[11] Burns S. E., Hassett J. P., Rossi M. V. (1997). Mechanistic Implications of the Intrahumic
Dechlorination of Mirex. Environ. Sci. Technol..

[12] Burns S. E., Hassett J. P., Rossi M. V. (1996). Binding
Effects on Humic-Mediated
Photoreaction: Intrahumic Dechlorination of Mirex in Water. Environ. Sci. Technol..

[13] Grandbois M., Latch D. E., Mcneill K. (2008). Microheterogeneous Concentrations
of Singlet Oxygen in Natural Organic Matter Isolate Solutions. Environ. Sci. Technol..

[14] Cheng K., Zhang L. Z., McKay G. (2023). Evaluating the Microheterogeneous
Distribution of Photochemically Generated Singlet Oxygen Using Furfuryl
Amine. Environ. Sci. Technol..

[15] Latch D. E., Stender B. L., Packer J. L., Arnold W. A., McNeill K. (2003). Photochemical
Fate of Pharmaceuticals in the Environment: Cimetidine and Ranitidine. Environ. Sci. Technol..

[16] Latch D. E., McNeill K. (2006). Microheterogeneity of Singlet Oxygen Distributions
in Irradiated Humic Acid Solutions. Science.

[17] Chu C., Lundeen R. A., Remucal C. K., Sander M., McNeill K. (2015). Enhanced Indirect
Photochemical Transformation of Histidine and Histamine through Association
with Chromophoric Dissolved Organic Matter. Environ. Sci. Technol..

[18] Kohn T., Grandbois M., McNeill K., Nelson K. L. (2007). Association with
Natural Organic Matter Enhances the Sunlight-Mediated Inactivation
of Ms2 Coliphage by Singlet Oxygen. Environ.
Sci. Technol..

[19] Wang H., Han M. Q., Wang M., Zhou H. X. (2022). Microheterogeneous
Triplet Oxidation of Hydrophobic Organic Contaminants in Dissolved
Black Carbon Solutions under Simulated Solar Irradiation. Environ. Sci. Technol..

[20] Zhou H. X., Yan S. W., Ma J. Z., Lian L. S., Song W. H. (2017). Development
of Novel Chemical Probes for Examining Triplet Natural Organic Matter
under Solar Illumination. Environ. Sci. Technol..

[21] Yan S. W., Sun J. Q., Sha H. T., Li Q., Nie J. X., Zou J. M., Chu C. H., Song W. H. (2021). Microheterogeneous
Distribution of Hydroxyl Radicals in Illuminated Dissolved Organic
Matter Solutions. Environ. Sci. Technol..

[22] Zhou H., Wang H., Wang H., Wang X., Ye Z., Hu X. (2025). Indirect Photodegradation
of Pharmaceutical and Personal Care Products
in Dissolved Black Carbon Solution: The Role of Microheterogeneous
Distribution of Hydroxyl Radical and Sorption. Water Res..

[23] Rosario-Ortiz F. L., Canonica S. (2016). Probe Compounds to
Assess the Photochemical Activity
of Dissolved Organic Matter. Environ. Sci. Technol..

[24] Li R., Zhao C., Yao B., Li D., Yan S. W., O’Shea K. E., Song W. H. (2016). Photochemical Transformation
of Aminoglycoside
Antibiotics in Simulated Natural Waters. Environ.
Sci. Technol..

[25] Sun L. N., Qian J. G., Blough N. V., Mopper K. (2015). Insights into the Photoproduction
Sites of Hydroxyl Radicals by Dissolved Organic Matter in Natural
Waters. Environ. Sci. Technol. Lett..

[26] Maizel A. C., Remucal C. K. (2017). Molecular Composition
and Photochemical Reactivity
of Size-Fractionated Dissolved Organic Matter. Environ. Sci. Technol..

[27] Romera-Castillo C., Chen M. L., Yamashita Y., Jaffe R. (2014). Fluorescence Characteristics
of Size-Fractionated Dissolved Organic Matter: Implications for a
Molecular Assembly Based Structure?. Water Res..

[28] Qu S., Kolodziej E. P., Cwiertny D. M. (2012). Phototransformation Rates and Mechanisms
for Synthetic Hormone Growth Promoters Used in Animal Agriculture. Environ. Sci. Technol..

[29] Kim I., Yu Z. Q., Xia B. H., Huang W. L. (2007). Sorption of Male
Hormones by Soils and Sediments. Environ. Toxicol.
Chem..

[30] Lee L. S., Strock T. J., Sarmah A. K., Rao P. S. C. (2003). Sorption and
Dissipation of Testosterone, Estrogens, and Their Primary Transformation
Products in Soils and Sediment. Environ. Sci.
Technol..

[31] Chun S., Lee J., Geyer R., White D. C. (2005). Comparison of Three Extraction Methods
for 17 Β-Estradiol in Sand, Bentonite, and Organic-Rich Silt
Loam. J. Environ. Sci. Health, Part B.

[32] Roh H., Chu K.-H. (2010). A 17β-Estradiol-Utilizing
Bacterium, Sphingomonas
Strain Kc8: Part I-Characterization and Abundance in Wastewater Treatment
Plants. Environ. Sci. Technol..

[33] Christl I., Ruiz M., Schmidt J. R., Pedersen J. A. (2016). Clarithromycin and
Tetracycline Binding to Soil Humic Acid in the Absence and Presence
of Calcium. Environ. Sci. Technol..

[34] Sibley S. D., Pedersen J. A. (2008). Interaction of the
Macrolide Antimicrobial Clarithromycin
with Dissolved Humic Acid. Environ. Sci. Technol..

[35] Young R. B., Latch D. E., Mawhinney D. B., Nguyen T. H., Davis J. C. C., Borch T. (2013). Direct Photodegradation
of Androstenedione and Testosterone
in Natural Sunlight: Inhibition by Dissolved Organic Matter and Reduction
of Endocrine Disrupting Potential. Environ.
Sci. Technol..

[36] Vulliet E., Falletta M., Marote P., Lomberget T., Païssé J.-O., Grenier-Loustalot M.-F. (2010). Light Induced
Degradation of Testosterone in Waters. Sci.
Total Environ..

[37] McNeill K., Canonica S. (2016). Triplet State Dissolved
Organic Matter in Aquatic Photochemistry:
Reaction Mechanisms, Substrate Scope, and Photophysical Properties. Environ. Sci. Process. Impacts.

[38] Brezová V., Šlebodová A., Staško A. (2009). Coffee as
a Source of Antioxidants: An Epr Study. Food
Chem..

[39] León-Carmona J.
R., Galano A. (2011). Is Caffeine
a Good Scavenger of Oxygenated Free Radicals?. J. Phys. Chem. B.

[40] Mezyk, S. P. ; Abud, E. M. ; Swancutt, K. L. ; McKay, G. ; Dionysiou, D. D. Removing Steroids from Contaminated Waters Using Radical Reactions. In Contaminants of Emerging Concern in the Environment: Ecological and Human Health Considerations; ACS Publications, 2010; pp 213–225.

[41] Li R., Zhao C., Yao B., Li D., Yan S., O’Shea K. E., Song W. (2016). Photochemical Transformation
of Aminoglycoside
Antibiotics in Simulated Natural Waters. Environ.
Sci. Technol..

[42] Yan S., Sun J., Sha H., Li Q., Nie J., Zou J., Chu C., Song W. (2021). Microheterogeneous Distribution of Hydroxyl Radicals
in Illuminated Dissolved Organic Matter Solutions. Environ. Sci. Technol..

[43] Mack J., Bolton J. R. (1999). Photochemistry of Nitrite and Nitrate in Aqueous Solution:
A Review. J. Photochem. Photobiol., A.

[44] Mondino E., Carena L., Gu C., Vione D. (2025). Photodegradation of
Pollutants in the Hydrophobic Cores of Dissolved Organic Matter: When
Is It Important?. Environ. Sci. Technol..

[45] Neale P. A., Escher B. I., Schäfer A. I. (2009). Ph Dependence
of Steroid Hormone—Organic
Matter Interactions at Environmental Concentrations. Sci. Total Environ..

[46] Neale P. A., Escher B. I., Schäfer A. I. (2008). Quantification
of Solute-Solute Interactions
Using Negligible-Depletion Solid-Phase Microextraction:: Measuring
the Affinity of Estradiol to Bulk Organic Matter. Environ. Sci. Technol..

[47] Yamamoto H., Liljestrand H. M., Shimizu Y., Morita M. (2003). Effects of Physical–
Chemical Characteristics on the Sorption of Selected Endocrine Disruptors
by Dissolved Organic Matter Surrogates. Environ.
Sci. Technol..

[48] Perminova I. V., Shirshin E. A., Konstantinov A. I., Zherebker A., Lebedev V. A., Dubinenkov I. V., Kulikova N. A., Nikolaev E. N., Bulygina E., Holmes R. M. (2018). The Structural
Arrangement and Relative
Abundance of Aliphatic Units May Effect Long-Wave Absorbance of Natural
Organic Matter as Revealed by (1)H Nmr Spectroscopy. Environ. Sci. Technol..

[49] Wang Z., Nagata M., Murano H., Pignatello J. J. (2024). Participation
of Strong Charge-Assisted Hydrogen Bonds in Interactions of Dissolved
Organic Matter Represented by Suwannee River Humic Acid. Water Res..

[50] Piccolo A. (2002). The Supramolecular
Structure of Humic Substances: A Novel Understanding of Humus Chemistry
and Implications in Soil Science. Adv. Agron..

[51] Smejkalová D., Piccolo A. (2008). Aggregation and Disaggregation
of Humic Supramolecular
Assemblies by Nmr Diffusion Ordered Spectroscopy (Dosy-Nmr). Environ. Sci. Technol..

[52] Korak J. A., McKay G. (2024). Critical Review of Fluorescence and Absorbance Measurements as Surrogates
for the Molecular Weight and Aromaticity of Dissolved Organic Matter. Environmental Science: Processes & Impacts.

[53] Hanson B., Wunsch U., Buckley S., Fischer S., Leresche F., Murphy K., D’Andrilli J., Rosario-Ortiz F. L. (2022). Dom Molecular
Weight Fractionation and Fluorescence Quantum Yield Assessment Using
a Coupled in-Line Sec Optical Property System. ACS ES&T Water.

[54] Hanson B., Wünsch U., Buckley S., Fischer S., Leresche F., Murphy K., D’Andrilli J., Rosario-Ortiz F. L. (2022). Dom Molecular
Weight Fractionation and Fluorescence Quantum Yield Assessment Using
a Coupled in-Line Sec Optical Property System. ACS ES&T Water.

[55] Page S. E., Sander M., Arnold W. A., McNeill K. (2012). Hydroxyl Radical
Formation
Upon Oxidation of Reduced Humic Acids by Oxygen in the Dark. Environ. Sci. Technol..

[56] Liu X., Pan Y., Yao Y., Chen S., Chen B., Chu C. (2025). Accelerated
Pollutant Degradation by Uv/H2o2 at the Air–Water Interface
of Microdroplets. Environ. Sci. Technol..

[57] Shu Z., Liu Q., Dai Z., Pan Z., Aeppli M., Wang Z. (2025). Heterogeneous
Photochemical Generation of Hydroxyl Radical in Mineral-Organics Systems:
Dual Roles of Iron Oxides. Environ. Sci. Technol..

[58] Grannas A. M., Pagano L. P., Pierce B. C., Bobby R., Fede A. (2014). Role of Dissolved
Organic Matter in Ice Photochemistry. Environ.
Sci. Technol..

