# Supplementary material for: Probing the Microheterogeneous Distribution of Photochemically Produced Hydroxyl Radicals in Dissolved Organic Matter
Source: Environ Sci Technol. 2025 Dec 17;60(1):1275–84. doi: 10.1021/acs.est.5c13430 (PMC12810377; doi:10.1021/acs.est.5c13430)
Supplement: Supplementary file 1 [file es5c13430_si_001.pdf]

Supporting Information for

Probing the Microheterogeneous Distribution of Photochemically Produced Hydroxyl Radicals in Dissolved Organic Matter

Kai Cheng[1] and Garrett McKay[1,*]

1. Zachry Department of Civil & Environmental Engineering, Texas A&M University, College Station, TX 77843, United States.

Corresponding author: 3136 TAMU, College Station, TX 77843; e-mail:gmckay@tamu.edu; phone: (979) 458-6540

Number of Pages: 18
Number of Text Sections: 6
Number of Tables: 1
Number of Figures: 10

Contents

Text S1. Solution preparation.................... 4
Text S2. Liquid phase extraction.................... 4
Text S3. HPLC analysis.................... 5
Text S4 Dialysis experiment.................... 5
Text S5. Photolysis kinetic modeling and sorption process modeling..................... 6
Text S6 Ultrafiltration.................... 7
Table S1. List of chemicals used in this study.................... 9
Figure S1. Absolute spectral irradiance profiles of the UV lamps used in this study for all photolysis experiments.................... 10
Figure S2. (A) Evaporation of experiment solution under 6 h irradiation and (B) temperature monitoring of experiment solution under 8 h irradiation..................... 10
Figure S3. (A) Extraction recovery of testosterone at different diethyl ether–to–sample volume ratios. (B) Recovery of testosterone compared to 17β-estradiol as a surrogate standard. Diethyl ether was used at ten times the sample volume for each extraction.................... 11
Figure S4. Validation of the reaction rate constant for testosterone with •OH. (A) Degradation of 10 μM testosterone in a solution containing 230 μM nitrite. (B) Degradation of 10 μM caffeine under identical conditions..................... 11
Figure S5. Phototransformation of testosterone (10 μM) mediated by •OH radicals generated from DOM photolysis under UV_{365}. DOM was used at 20 mg/L and buffered with 10 mM phosphate at pH 7. 17β-estradiol was included as a surrogate to monitor liquid-phase extraction recovery, shown as gray squares. Green dots correspond to aquatic-derived DOM samples: (A) SRHA, (B) SRFA, (C) SRNOM, and (D) MRNOM. Yellow dots indicate terrestrial-derived DOM: (E) PPHA, (F) PPFA, (G) ESHA, and (H) ESFA..................... 12
Figure S6. Sorption isotherms of testosterone to DOM isolates, determined using dialysis (1K Da membrane cutoff). DOM concentration was 100 mg/L, and testosterone was spiked at 1–50 μM. Equilibrium was reached after 72 hours. Linear fitting between C_w and C_d was used to calculate partitioning coefficients. Dashed gray lines represent the 95% confidence intervals of the regression. Green dots denote aquatic-derived DOM: (A) SRHA, (B) SRFA, (C) SRNOM, and (D) MRNOM. Yellow dots represent terrestrial-derived DOM: (E) PPHA, (F) PPFA, (G) ESHA, and (H) ESFA.................... 13
Figure S7. Correlation analysis between testosterone partitioning coefficient (log K_{OC}) and DOM compositional characteristics: (A) oxygen-to-carbon ratio (O/C), (B) aliphalicity, (C) aromaticity, and (D) phenolic content. Each parameter was obtained from IHSS-reported characterization of the DOM isolates..................... 14
Figure S8. Comparison of $[^{\bullet}OH]_{DOM}/[^{\bullet}OH]_{aq}$ ratios as a function of DOM composition. (C) shows the relationship with aromaticity and (D) with aliphaticity. DOM compositional values were sourced from IHSS documentation. $[^{\bullet}OH]_{DOM}/[^{\bullet}OH]_{aq}$ ratios were determined using both testosterone and benzoate probes. DOM isolates ESHA and ESFA were excluded due to inconsistencies in probe responses..................... 15
Figure S9. Testosterone sorption and photodegradation in high molecular weight (>3 kDa) DOM fractions. (A) and (B) show sorption isotherms of testosterone to >3 kDa fractions of SRHA and SRNOM, respectively, using a DOM concentration of 50 mg C/L and testosterone spiked from 1 to 45 μM. (C) and (D) illustrate the photodegradation of testosterone (10 μM) by

$^{\bullet}$OH generated from the same >3 kDa fractions of SRHA and SRNOM, respectively, at 10 mgC/L DOM buffered with 10 mM phosphate at pH 7. 17β-estradiol was added as a surrogate to evaluate the recovery efficiency of testosterone during extraction..................................... 16

Figure S10. UV–vis absorbance spectra of bulk and fractionated (>3 kDa) DOM. (A) SRHA and (B) SRNOM. 17

Reference:... 18

Text S1. Solution preparation

Lab grade water (18.2 MΩ-cm resistivity) was used for all water-based solution preparation. DOM stock solutions (~200 mg/L) were prepared by dissolving the solid isolate in lab grade water produced from a Barnstead Nanopure purification system (Thermo Scientific, 18.2 MΩ-cm resistivity). NaOH (1 M) was added incrementally until a pH ~7 was reached. Following 12 hours of stirring in the dark, the 0.45 μm syringe filters (polyethersulfone, VWR) were pre-rinsed with over 50 mL of lab-grade water before use. The stock solutions were then filtered and stored in amber bottles at 4°C shielded from light.

For the quantification of •OH in aqueous bulk phase, benzoate and terephthalate were selected as the probe compounds that react with •OH leading to the formation of hydroxylated products. The preparation of the solution and the setup for instrumental analysis can be found in detail from our previously published work.[1]

Text S2. Liquid phase extraction

A control experiment was conducted to evaluate the difference between testosterone concentrations measured by direct HPLC analysis and those obtained after liquid-liquid extraction. The same amount of testosterone was added to ultrapure water and a 20 mg/L SRHA solution, yielding an initial measured concentration of 9.85 μM. After 24 h of equilibration ($n = 5$), the testosterone concentration in water was 9.68 ± 0.30 μM, while that in the SRHA solution was 8.80 ± 0.18 μM, indicating a small sorbed fraction. The observed difference reflects testosterone sorption to DOM, as direct HPLC detects only the freely dissolved fraction. Although not all DOM isolates were tested, the sorbed fraction was minor relative to the dissolved concentration; however, excluding it could bias the apparent •OH estimation. Therefore, liquid-liquid extraction was used to improve quantification accuracy.

It is important to minimize the variation at the extraction stage to maintain extraction efficiency and reproducibility. Testosterone recovery was targeted near 100% to ensure reliable instrumental quantification. As shown in Figure S2, we optimized the extraction volume and

validated estradiol's effectiveness as a surrogate for testosterone recovery. At a 10:1 diethyl ether-to-sample volume ratio, testosterone recovery reached 96.1%. By increasing to 20:1 diethyl ether-to-sample volume ratio, testosterone recovery increased to 98.1%. Estradiol showed comparable recovery efficiency, indicating its suitability as a surrogate for testosterone extraction. Therefore, a 3 mL aliquot of sample volume was withdrawn during DOM photolysis. A 5 μL aliquot of estradiol solution (2 mM) was added as a surrogate. Subsequently, the solution was mixed with 30 mL diethyl ether, vortexed for 5 min, and transferred to a separatory funnel. The mixture was allowed to settle undisturbed for 30 minutes to enable phase separation. The aqueous layer was drained into a vial, and the diethyl ether phase was evaporated under airflow. After evaporation, the extracted testosterone and estradiol were redissolved in 5 mL of solvent consisting of 50% methanol and 50% water. The concentration of testosterone and estradiol were analyzed by HPLC. For quality control, the aqueous phase (drained to the vial) was analyzed as well.

Text S3. HPLC analysis

The concentration of testosterone was monitored for its loss by a UV detector. The concentration of estradiol as surrogate was monitored by a fluorescent detector.

<table>
<tr><th>Parameter</th><th>Testosterone</th><th>Estradiol</th></tr>
<tr><td>Column</td><td colspan="2">C18</td></tr>
<tr><td>Mobile Phase</td><td colspan="2">40% H_2O and 60% acetonitrile</td></tr>
<tr><td>Flow Rate</td><td colspan="2">1 mL/min</td></tr>
<tr><td>Temperature</td><td colspan="2">30 °C</td></tr>
<tr><td>Injection volume</td><td colspan="2">50 μL</td></tr>
<tr><td>UV wavelength</td><td>250 nm</td><td></td></tr>
<tr><td>Fluorescent excitation wavelength</td><td>—</td><td>280 nm</td></tr>
<tr><td>Fluorescent emission wavelength</td><td>—</td><td>344 nm</td></tr>
<tr><td>Elution Time</td><td>3.4 min</td><td>3.0 min</td></tr>
</table>

*Ascentis C18 selectivity column (15 cm × 4.6 mm, 5 μm, Supelco).

Text S4 Dialysis experiment

Dialysis tubing (Spectra/Por 7, 1 kDa molecular weight cutoff) was rinsed and soaked overnight to remove residual NaN_3. A 10 mL aliquot of 100 mg/L DOM solution (pH 7) was

loaded into the tubing, sealed with polypropylene closures, and placed into a 100 mL amber bottle containing 50 mL of 10 mM phosphate buffer for predialysis over 24 hours. Afterward, the external buffer was replaced with 20 mL of testosterone solution (1–50 μM, 10 mM phosphate buffer). The containers were gently shaken in the dark at 25 °C for 72 hours to ensure sorption equilibrium. Testosterone concentrations in the external solution before and after sorption process were analyzed by mass balance to determine partition coefficient.

Considering that DOM is a dispersed organic phase, the partitioning of testosterone to DOM can be described using a simple linear-phase partitioning model, as shown.[2, 3]

$$TT + DOM \rightleftharpoons TT \cdot DOM \qquad (S1)$$

where TT stands for testosterone, and the fraction of testosterone associated with DOM is determined by the partitioning coefficient, $K_{OC}, L/kg_C$, where was determined from the equation below:

$$K_{OC}^{TT} = \frac{C_{DOM}^{TT}}{C_w^{TT}} \qquad (2)$$

where $C_{DOM}^{TT}(mol/kg_C)$ represents the sorbed concentration of testosterone with a unit of, and C_w^{TT} stands for the testosterone in aqueous phase with a concentration of mol/L.

Text S5. Photolysis kinetic modeling and sorption process modeling.

Through the HPLC analysis, the kinetic rate of testosterone loss can be determined as a function of time. The degradation of testosterone followed the pseudo-first-order kinetic expressed as eq. S3:

$$ln\frac{[TT]}{[TT]_0} = -k_2^{TT}[OH]_{app}^{TT} \cdot [TT] = -k_{obs}^{TT} \cdot [TT] \qquad (S3)$$

In the expression, $[TT]$ represents the concentration of testosterone (in the unit of *M*), k_{obs}^{TT} represents the pseudo-first order rate constant (in the unit of s^{-1}) determined from performing linear fitting, k_2^{TT} represents the second-order rate constant ($M^{-1}s^{-1}$) of the testosterone with $^{\bullet}$OH, and $[OH]_{app}^{TT}$ stands for the apparent concentration of $^{\bullet}$OH experienced by testosterone,

which can be divided into two fractions: the •OH in the DOM phase that's experienced by the fraction of testosterone (f_{DOM}) partitioned into the DOM phase, and the •OH in the bulk phase that's experienced by the fraction of testosterone (f_{aq}) in aqueous solution. Mathematically, $[OH]_{app}^{TT}$ can be described as eq. S4:

$$[OH]_{app}^{TT} = f_{DOM} \cdot [OH]_{DOM}^{TT} + f_{aq} \cdot [OH]_{aq}^{TT} \qquad (S4)$$

In this equation, $[OH]_{DOM}^{TT}$ is the concentration of •OH in the DOM phase, and $[OH]_{aq}^{TT}$ is the concentration of •OH in the aqueous phase.

The fraction of testosterone bound to DOM (f_{DOM}) was modeled as eq. S4.

$$f_{DOM} = \frac{K_{DOM}^{TT} \cdot [DOM]}{1 + K_{DOM}^{TT} \cdot [DOM]} \qquad (S5)$$

And the fraction of testosterone in aqueous phase (f_{aq}) is given by eq. 6:

$$f_{aq} = 1 - f_{DOM} \qquad (S6)$$

Therefore, the concentration of •OH in the DOM phase ($[OH]_{DOM}^{TT}$) can be determined.

Using SRHA as an example, a concentration of 20 mg/L was used in the photolysis experiments. The carbon content of SRHA is reported as 54.59% by the IHSS, corresponding to 10.918 mg_C/L. From the dialysis experiment, the sorption coefficient of testosterone to SRHA was determined to be 5145 ± 441 L/kg_C. Therefore, after 48 h of equilibration, the fraction of testosterone sorbed to 20 mg/L (10.918 mg_C/L) SRHA was calculated using Eq. S5 as 0.053±0.005. As noted in the manuscript, the small fraction of sorbed testosterone contributes negligibly to •OH quenching relative to DOM itself. Thus, the benzoate/terephthalate-derived $[^{\bullet}OH]_{aq}$ values were used in Eq. S4 to calculate [•OH] in the DOM phase.

Text S6 Ultrafiltration

Ultrafiltration was performed using a 200 mL stirred cell (UFSC20001, Amicon) equipped with a 3 kDa molecular weight cutoff membrane. The membrane was pre-cleaned by immersion in nanopure water with constant stirring for 1 hour, with the water replaced every 15 minutes.

After rinsing, the membrane was installed on the supporting disc below the apparatus cylinder, and a nitrogen gas line was connected to maintain a pressure below 75 psi. A final flush using 100 mL of nanopure water was passed through the membrane before using the DOM solutions for ultrafiltration, and the total organic carbon (TOC) of the permeate using nanopure water was also monitored to ensure a complete cleaning of the membrane. DOM solutions (SRHA and SRNOM, ~150 mg/L, pH 7) were then processed by adding 200 mL of solution to the ultrafiltration chamber. Permeate was collected when 100 mL of DOM solution was pushed through the membrane. Then 100 mL nanopure was added to the retentate to collect a second batch of permeate. This is to ensure a more complete separation of the DOM molecules with different molecular weights. Both the retentate and permeate (two batches) were collected for analysis, including TOC (analyzed by total organic analyzer, TOC-L series, Shimadzu), UV-visible spectroscopy. The retentate and the first batch of permeate were used for photolysis experiment. The retentate fractions were also tested for their interaction with testosterone.

The 3 kDa ultrafiltration membrane cutoff was selected based on the molecular-weight characterization of SRFA reported by McAdams et al.[4], in which SRFA exhibited a weight-average molecular weight of approximately 2.0-2.3 kDa. SRHA, by contrast, is known to possess a higher molecular weight. In this study, we sought to evaluate how molecular-weight heterogeneity influences $^{\bullet}$OH microheterogeneity, given that the compositional analysis (Figure 3 in manuscript) indicated a negative correlation between $^{\bullet}$OH distribution and aromaticity, and a positive correlation with aliphalicity. Therefore, a membrane cutoff slightly higher than the reported weight-average molecular weight of SRFA (3 kDa) was chosen to effectively separate DOM into high- and low-molecular-weight fractions, enriching the higher-aromaticity components in the retentate. Although only two isolates were selected for size fractionation, SRNOM is one of the most widely studied isolate materials and results from this sample are expected to be applicable to other DOM isolates.

Table S1. List of chemicals used in this study.

Chemical/Material	CAS Number	Purity (%)	Supplier	Purpose/Use
Testosterone	58-22-0	≥98	Sigma-Aldrich	Probe compound for OH quantification
17β-Estradiol	50-28-2	≥98	Sigma-Aldrich	Recovery surrogate for testosterone
Diethyl ether	60-29-7	≥99.5	Sigma-Aldrich	Extraction solvent for testosterone
Methanol	67-56-1	HPLC grade	Fisher Scientific	Organic solvent for HPLC analysis
Acetonitrile	75-05-8	HPLC grade	Fisher Scientific	Organic solvent for HPLC analysis
Potassium dihydrogen phosphate	7778-77-0	≥99	Sigma-Aldrich	Component of phosphate buffer (pH 7)
Dipotassium hydrogen phosphate	7758-11-4	≥99	Sigma-Aldrich	Component of phosphate buffer (pH 7)
Benzoic acid	65-85-0	≥99.5	Sigma-Aldrich	Hydrophilic OH probe
Terephthalic acid	100-21-0	≥98	Sigma-Aldrich	Hydrophilic OH probe
Dialysis tubing (1 kDa cutoff)	N/A	N/A	Spectra/Por 7	Partitioning experiment
Ultrafiltration membrane (3 kDa)	N/A	N/A	Millipore	Molecular weight fractionation

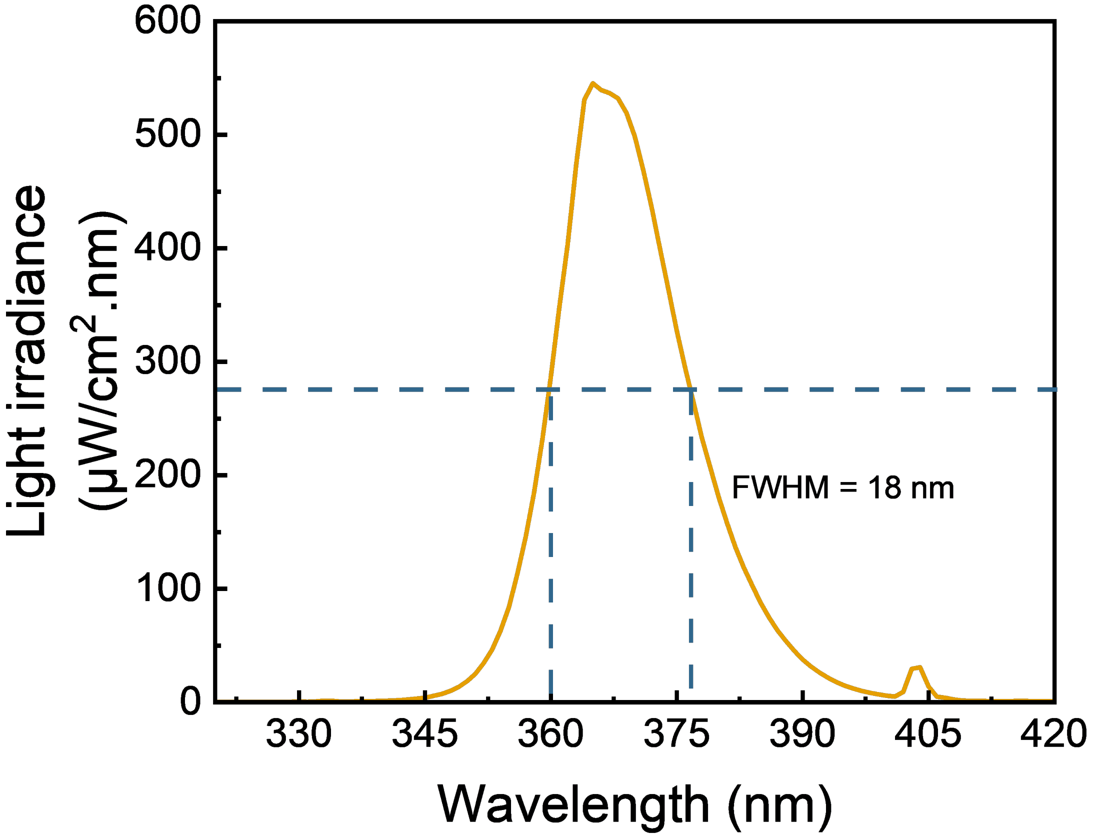

Figure S1. Absolute spectral irradiance profiles of the UV lamps used in this study for all photolysis experiments.

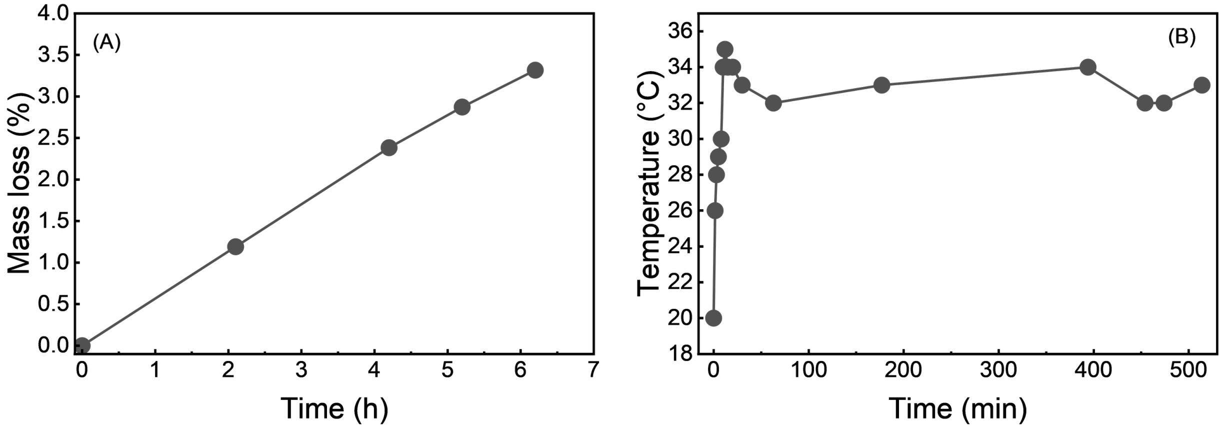

Figure S2. (A) Evaporation of experiment solution under 6 h irradiation and (B) temperature monitoring of experiment solution under 8 h irradiation.

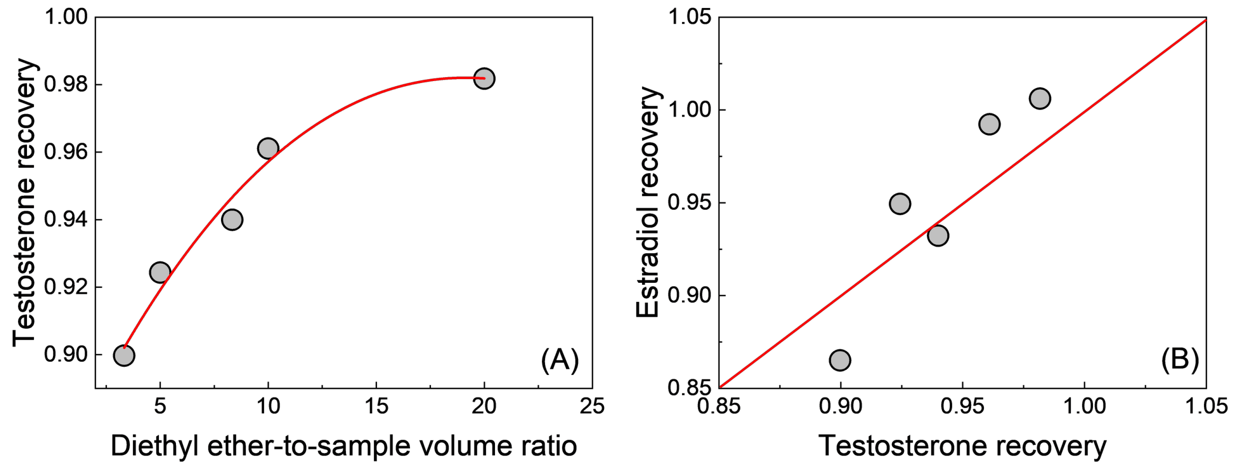

Figure S3. (A) Extraction recovery of testosterone at different diethyl ether–to–sample volume ratios. (B) Recovery of testosterone compared to 17β-estradiol as a surrogate standard. Diethyl ether was used at ten times the sample volume for each extraction.

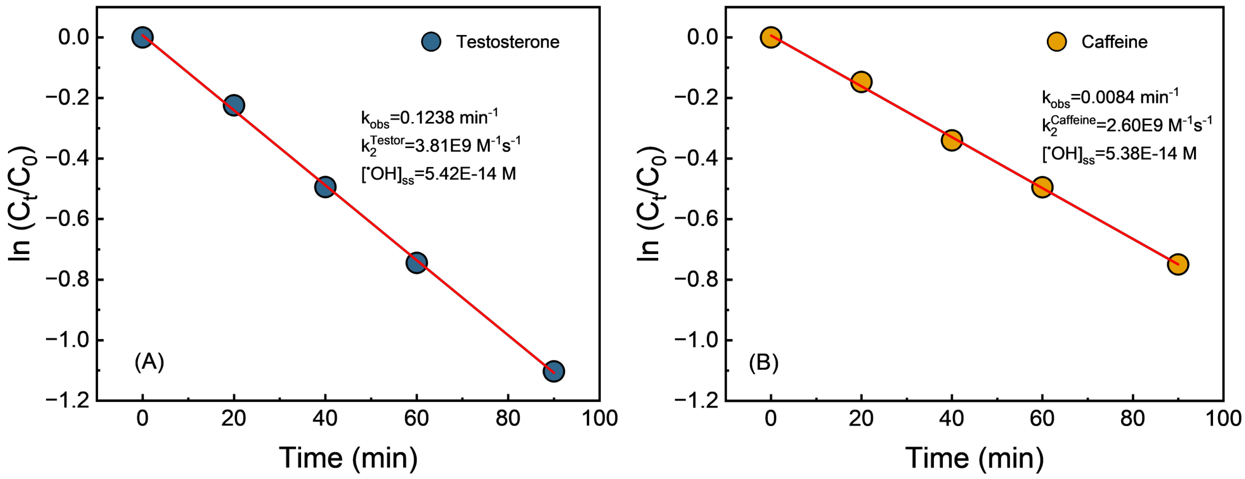

Figure S4. Validation of the reaction rate constant for testosterone with $^{\bullet}$OH. (A) Degradation of 10 μM testosterone in a solution containing 230 μM nitrite. (B) Degradation of 10 μM caffeine under identical conditions.

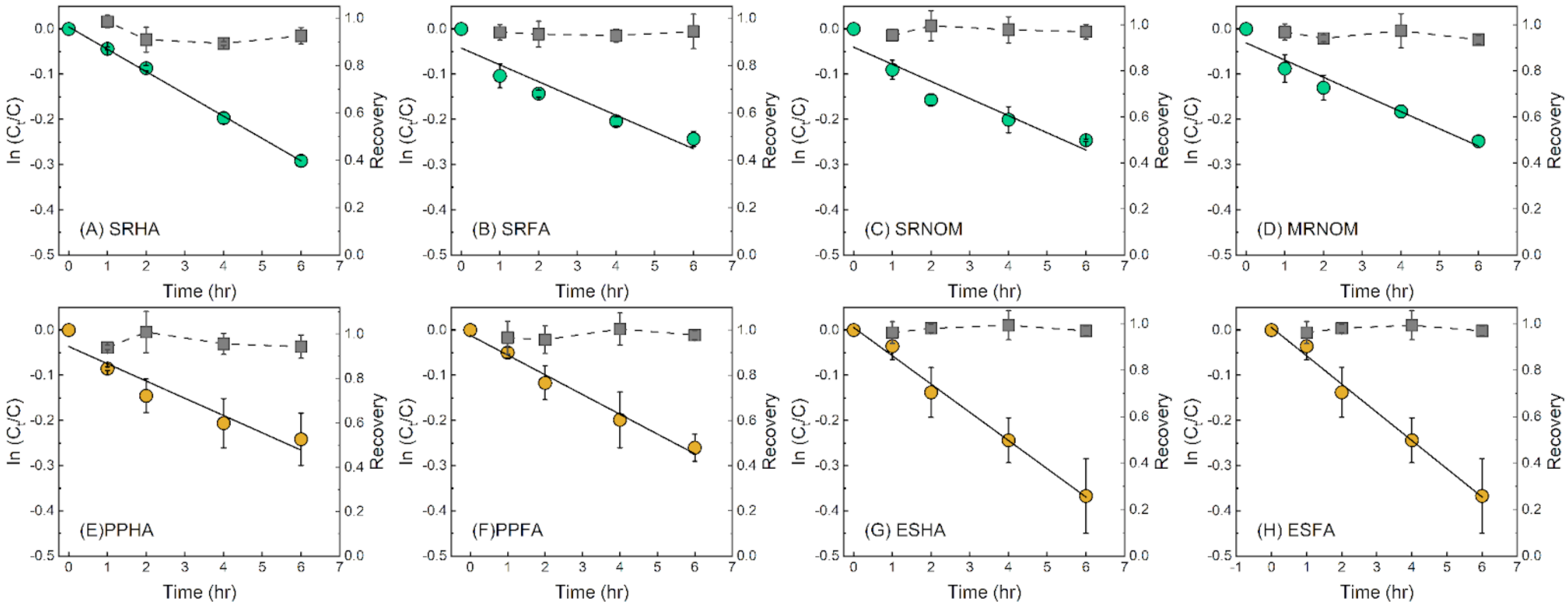

Figure S5. Phototransformation of testosterone (10 μM) mediated by •OH radicals generated from DOM photolysis under UV$_{365}$. DOM was used at 20 mg/L and buffered with 10 mM phosphate at pH 7. 17β-estradiol was included as a surrogate to monitor liquid-phase extraction recovery, shown as gray squares. Green dots correspond to aquatic-derived DOM samples: (A) SRHA, (B) SRFA, (C) SRNOM, and (D) MRNOM. Yellow dots indicate terrestrial-derived DOM: (E) PPHA, (F) PPFA, (G) ESHA, and (H) ESFA.

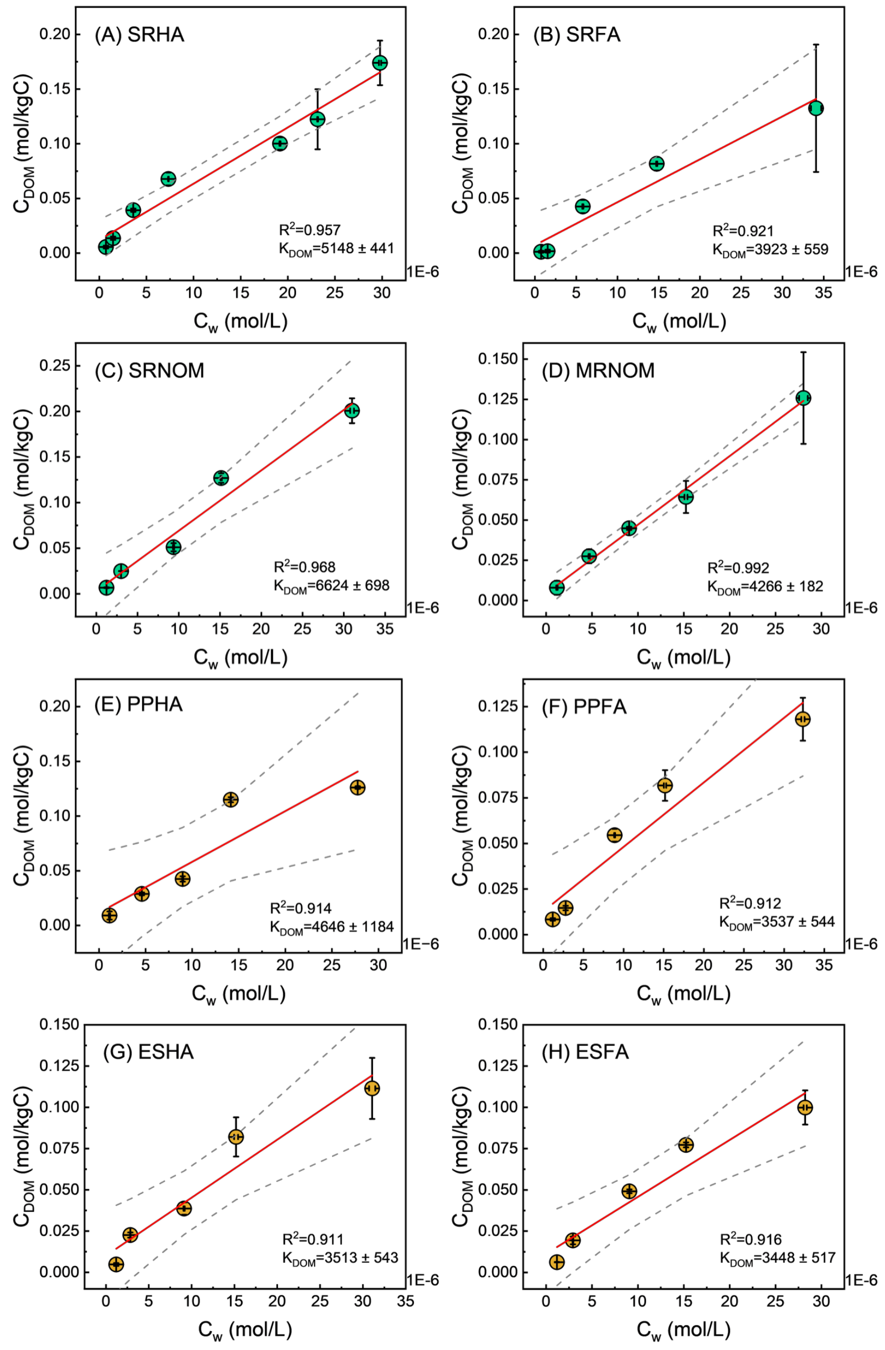

Figure S6. Sorption isotherms of testosterone to DOM isolates, determined using dialysis (1K Da membrane cutoff). DOM concentration was 100 mg/L, and testosterone was spiked at 1–50 μM. Equilibrium was reached after 72 hours. Linear fitting between C_w and C_d was used to calculate partitioning coefficients. Dashed gray lines represent the 95% confidence intervals of the regression. Green dots denote aquatic-derived DOM: (A) SRHA, (B) SRFA, (C) SRNOM, and (D) MRNOM. Yellow dots represent terrestrial-derived DOM: (E) PPHA, (F) PPFA, (G) ESHA, and (H) ESFA.

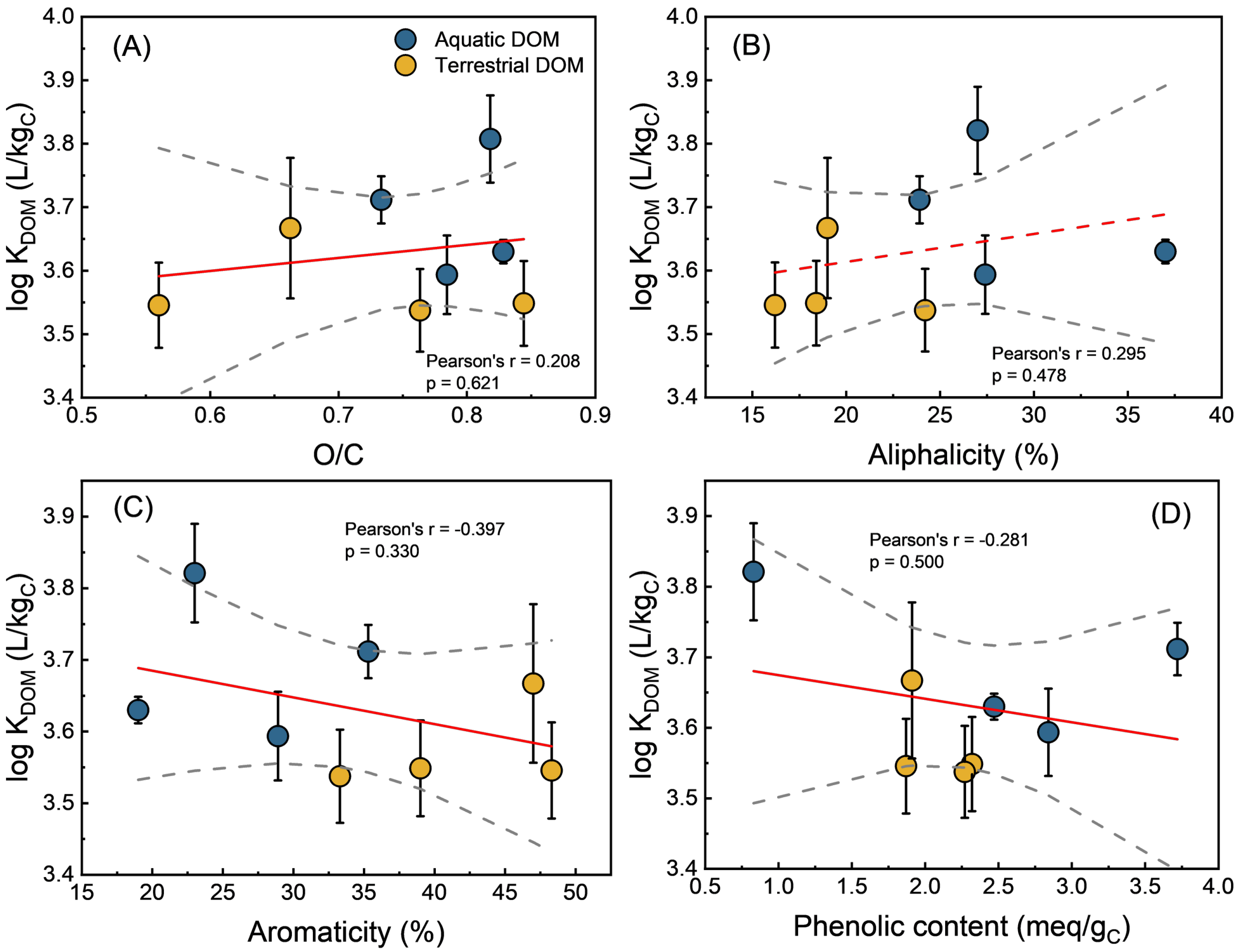

Figure S7. Correlation analysis between testosterone partitioning coefficient (log K_{OC}) and DOM compositional characteristics: (A) oxygen-to-carbon ratio (O/C), (B) aliphalicity, (C) aromaticity, and (D) phenolic content. Each parameter was obtained from IHSS-reported characterization of the DOM isolates.

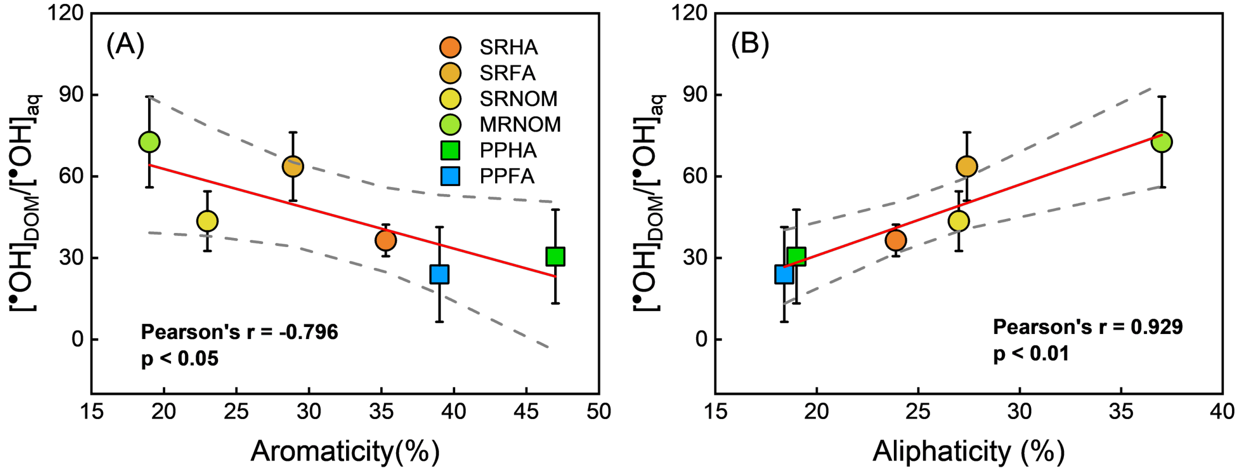

Figure S8. Comparison of $[^{\bullet}OH]_{DOM}/[^{\bullet}OH]_{aq}$ ratios as a function of DOM composition. (C) shows the relationship with aromaticity and (D) with aliphaticity. DOM compositional values were sourced from IHSS documentation. $[^{\bullet}OH]_{DOM}/[^{\bullet}OH]_{aq}$ ratios were determined using both testosterone and benzoate probes. DOM isolates ESHA and ESFA were excluded due to inconsistencies in probe responses.

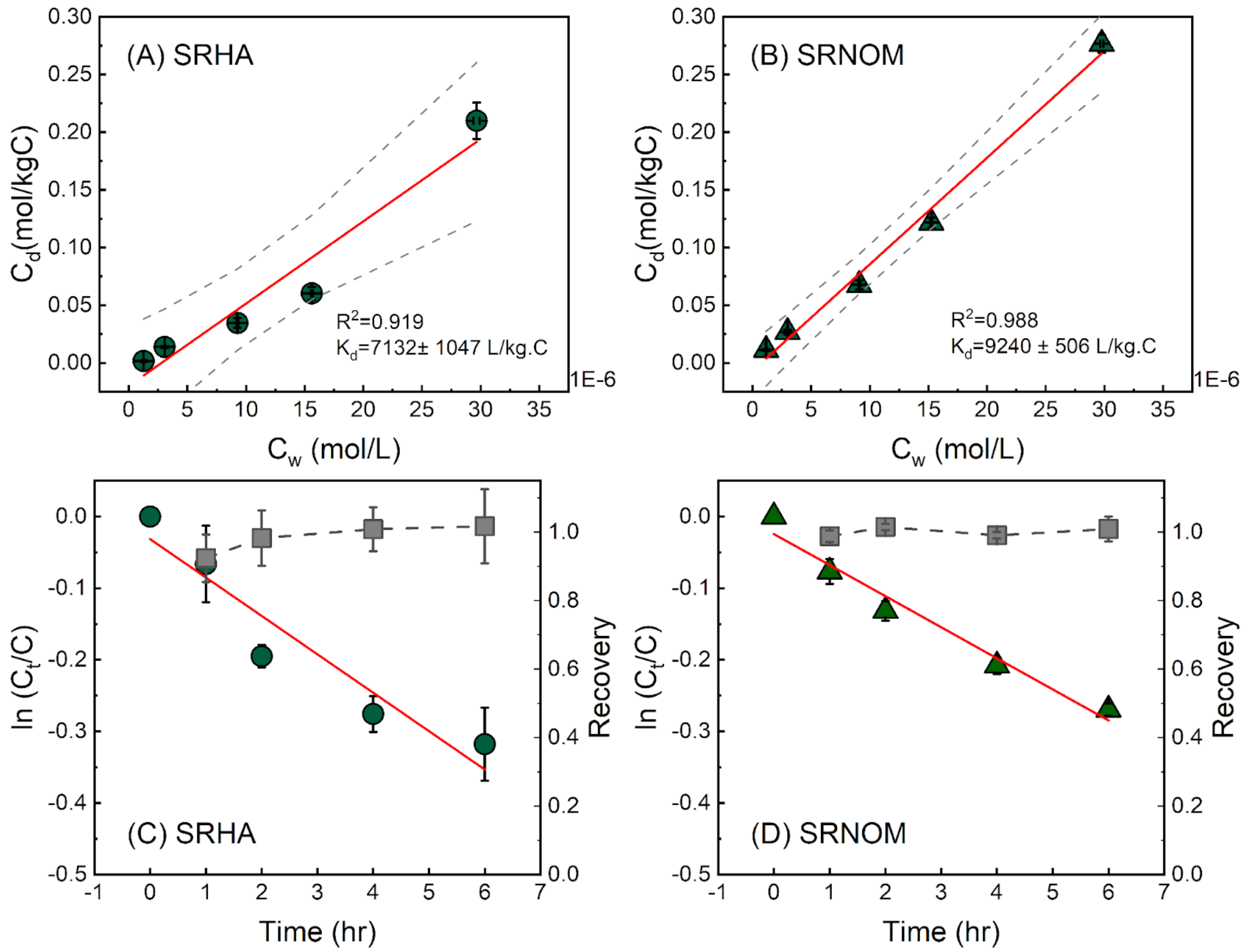

Figure S9. Testosterone sorption and photodegradation in high molecular weight (>3 kDa) DOM fractions. (A) and (B) show sorption isotherms of testosterone to >3 kDa fractions of SRHA and SRNOM, respectively, using a DOM concentration of 50 mg C/L and testosterone spiked from 1 to 45 μM. (C) and (D) illustrate the photodegradation of testosterone (10 μM) by •OH generated from the same >3 kDa fractions of SRHA and SRNOM, respectively, at 10 mgC/L DOM buffered with 10 mM phosphate at pH 7. 17β-estradiol was added as a surrogate to evaluate the recovery efficiency of testosterone during extraction.

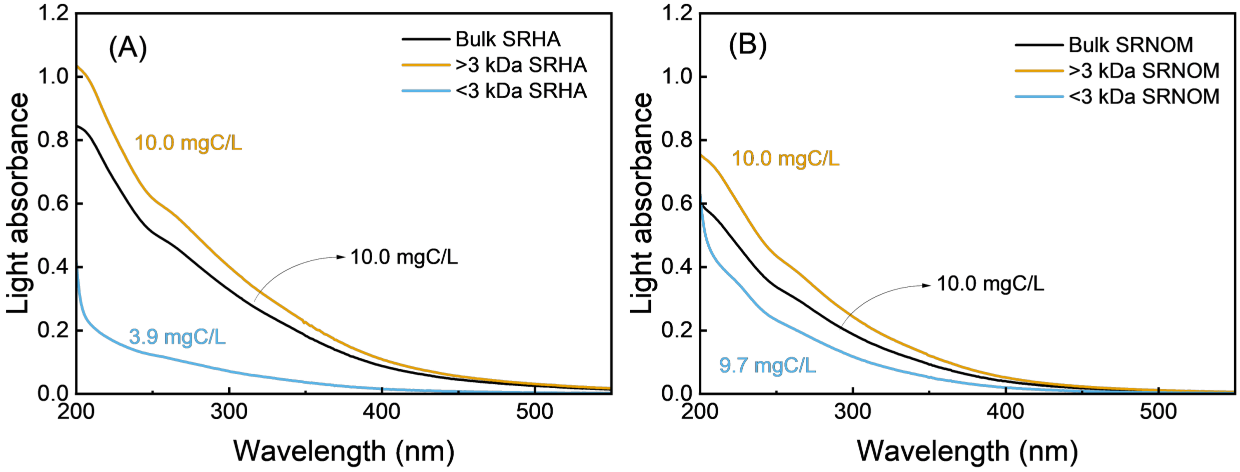

Figure S10. UV–vis absorbance spectra of bulk and fractionated (>3 kDa) DOM. (A) SRHA and (B) SRNOM.

Reference:

(1) Cheng, K.; Li, H.; Laszakovits, J. R.; Sharpless, C. M.; Rosario-Ortiz, F.; McKay, G. Probing the Photochemical Formation of Hydroxyl Radical from Dissolved Organic Matter: Insights into the H2O2-Dependent Pathway. *Environ Sci Technol* **2025**.

(2) Grandbois, M.; Latch, D. E.; Mcneill, K. Microheterogeneous Concentrations of Singlet Oxygen in Natural Organic Matter Isolate Solutions. *Environ Sci Technol* **2008**, *42* (24), 9184-9190. DOI: 10.1021/es8017094.

(3) Yan, S. W.; Sun, J. Q.; Sha, H. T.; Li, Q.; Nie, J. X.; Zou, J. M.; Chu, C. H.; Song, W. H. Microheterogeneous Distribution of Hydroxyl Radicals in Illuminated Dissolved Organic Matter Solutions. *Environ Sci Technol* **2021**, *55* (15), 10524-10533. DOI: 10.1021/acs.est.1c03346.

(4) McAdams, B. C.; Aiken, G. R.; McKnight, D. M.; Arnold, W. A.; Chin, Y. P. High Pressure Size Exclusion Chromatography (HPSEC) Determination of Dissolved Organic Matter Molecular Weight Revisited: Accounting for Changes in Stationary Phases, Analytical Standards, and Isolation Methods. *Environ Sci Technol* **2018**, *52* (2), 722-730. DOI: 10.1021/acs.est.7b04401 From NLM Medline.
